# Multi-objective optimization of national dietary guidelines: balancing nutrition, environment, and economy

**DOI:** 10.3389/fnut.2026.1758724

**Published:** 2026-03-26

**Authors:** Yehao Hu, Fenghui Zhao, Mengmeng Zhang, Tingting Wang, Han Li, Tong Fu, Tengyun Gao, Chuanyou Su

**Affiliations:** Henan International Joint Laboratory of Nutrition Regulation and Ecological Raising of Domestic Animal, College of Animal Science and Technology, Henan Agricultural University, Zhengzhou, China

**Keywords:** climate-smart policy, cost-effectiveness analysis, decision-support methods, food-based recommendations, micronutrient adequacy, sustainable diets

## Abstract

**Background:**

National dietary guidelines have largely overlooked the environmental sustainability of food systems. A multi-objective optimization framework to evaluate trade-offs among nutritional requirements, greenhouse-gas emissions, and economic costs across four countries at different development stages—the United States, China, Australia, and New Zealand.

**Methods:**

Using epidemiologically informed nutrient constraints and life-cycle environmental data, we systematically evaluate dietary scenarios that optimize animal-product consumption while ensuring essential nutrient adequacy.

**Results:**

Our analysis shows that strategic reductions in beef consumption can lower diet-related greenhouse-gas emissions by 28–62%, while chicken and eggs are reallocated to maintain nutritional integrity. Economic impacts vary markedly: vitamin-optimized scenarios reduce costs by 23% in China but increase costs by 19% in Australia.

**Conclusion:**

The results reveal distinct optimization pathways that necessitate tailored policy measures. This framework enables policymakers to revise food-based dietary guidelines to align with climate commitments while safeguarding nutritional security and economic viability across diverse national contexts.

## 1 Introduction

The global food system is facing an acute crisis in relation to resources and the environment (1, 2), which poses a threat to both human health and ecological stability (3). Excessive waste and inefficient consumption of resources in order to meet population growth demands not only have a detrimental effect on public health, but also result in a continuously rising environmental footprint with potentially irreversible consequences for the availability of natural resources and the integrity of ecosystems (4, 5). It is therefore vital that national food guidelines adopt a comprehensive approach, integrating food system resource efficiency, environmental health, and human nutrition. This is the only way to achieve sustainable and healthy diets under diverse policy contexts (6).

The significance of national food-based dietary guidelines in public health is indisputable (7, 8); they serve as a crucial bridge between nutritional science and practical application. These guidelines aim to reduce diet-related non-communicable diseases by translating scientific knowledge into actionable recommendations (6). A substantial body of epidemiological evidence has been amassed from three major sources: the Nurses' Health Study, the Health Professionals Follow-up Study (9), and the European Prospective Investigation into Cancer and Nutrition (10). Consistently demonstrates that diets emphasizing plant foods, coupled with a moderate intake of animal products, significantly reduce the risk of cardiovascular disease, type 2 diabetes, and certain cancers (11, 12). Given the persistent role of animal-derived foods in these balanced diets, optimizing their production becomes essential. Emerging evidence from ruminant microbiome research reveals the mechanistic basis for such optimization. This study optimizes human dietary patterns—specifically the intake of animal-source foods (ASF)—to meet nutrient requirements while reducing environmental and economic burdens. Production-side interventions, including targeted improvements in ruminant feeding strategies, can enhance nutrient-use efficiency and lower emission intensities in some systems (13, 14). However, relying solely on production-side technological improvements is insufficient to meet climate targets. These innovations must be coupled with demand-side dietary shifts to avoid reinforcing unsustainable industrial practices. This provides a dual rationale: improving the biological efficiency of necessary animal products while simultaneously optimizing their consumption levels within dietary frameworks.

Animal products are a valuable source of essential nutrients. However, the levels of these nutrients recommended by numerous countries are higher than the global recommended levels (15). This overconsumption contributes substantially to diet-related disease burden, while the production of animal agriculture generates approximately 12% of anthropogenic greenhouse gas emissions, creating a dual imperative for dietary guideline reform. In high-income countries, such as the United States, Australia, and several European nations, there is an increasing tendency to advocate for plant-forward or planetary health diets (16). These recommended consumption patterns focus on minimizing the intake of red and processed meats while significantly increasing the proportion of legumes, nuts, and whole grains to meet both health and environmental targets (17, 18). It is evident from the extant research that plant-based foodstuffs generally demonstrate a lower degree of resource intensity and environmental impact in comparison with animal products (19).

Contemporary dietary guidelines, which are predominantly informed by the nutrition adequacy paradigm, frequently overlook systemic resource efficiency and economic implications (20). Livestock farming requires 2–25 times more natural resources than plant production (21). Notwithstanding the advent of frameworks such as the EAT-Lancet Commission, which have been developed with the intention of incorporating sustainability principles (22). The majority of dietary guidelines derived from food-based sources have thus far neglected to incorporate resource conservation and circularity considerations into their recommendations in a systematic manner.

The present study addresses three critical gaps in sustainable nutrition policy through the application of a multicountry optimization framework across the United States, China, Australia, and New Zealand. The present study aims to build upon the epidemiological foundation which establishes food-based dietary guidelines as consumer-oriented health tools (8, 23, 24), An investigation was conducted into the trade-offs between micronutrient adequacy and emissions reduction when optimizing animal product intake. Furthermore, the cost differences affecting the economic feasibility of sustainable diets under different economic environments were analyzed. Finally, policy mechanisms for reforming dietary guidelines while ensuring high-quality nutrition as much as possible were identified. This approach has the capacity to identify dietary transition pathways for specific situations, thereby simultaneously promoting human health, resource conservation, and socioeconomic equity.

## 2 Methods

### 2.1 Study design and country selection

The present study employed a strategic comparative case study approach across four countries: the United States, China, Australia, and New Zealand. The selection of the country was grounded in three transparent, methodologically rigorous criteria. Firstly, it is evident that each country maintains publicly accessible, government-endorsed food-based dietary guidelines that provide standardized recommendations for population-level dietary intake. The study utilized the government-endorsed food-based dietary guidelines (FBDGs) for each nation that were current at the time of the analysis. Specifically, we extracted recommendations from the Dietary Guidelines for Americans 2020–2025 (US. Department of Agriculture), the Dietary Guidelines for Chinese Residents (Chinese Nutrition Society), the Australian Dietary Guidelines Summary (National Health and Medical Research Council), and the Eating and Activity Guidelines (Health New Zealand). Secondly, comprehensive datasets were available for population dietary intake patterns, nutrient consumption profiles, and health outcomes necessary for robust optimisation modeling. Thirdly, reliable food pricing data and greenhouse gas emission factors specific to national food supply chains were accessible, enabling integrated sustainability analysis. A detailed summary of data sources, including the specific years and issuing institutions for dietary intake data (Supplementary Table S1), nutrient composition databases (Supplementary Table S2), greenhouse gas emission factors (Supplementary Table S4), and producer price indices, is provided in the Supplementary materials.

The analytical framework incorporated five archetypal scenarios designed to address overnutrition challenges and examine sustainable dietary transition pathways. These scenarios integrated major food groups as defined within each nation's existing food-based dietary guidelines, ensuring policy relevance and practical applicability (see Supplementary Table S1). Country selection adhered to rigorous transparency criteria, including food-based dietary guideline availability, comprehensive population health and dietary profile data, and sufficient statistical coverage for robust analytical modeling (25).

While our empirical analysis focuses on four economies, the workflow—data harmonization, scenario construction, multi-objective trade-off analysis, and uncertainty assessment—can be applied to other settings. However, the quantitative results should not be extrapolated without country-specific inputs, including an overview of population-related nutritional needs and health risks, locally observed dietary patterns and feasible alternatives, as well as costs and emissions in specific circumstances (26).

### 2.2 The process of converting food types in dietary guidelines into the daily diet

The national food-based dietary guideline recommendations were systematically translated into quantified daily diets via FAO food balance sheets and standardized conversion matrices (27) (see Supplementary materials p. 2, Supplementary Table S2). Broad FBDG food groups (e.g., grains, red meat) were mapped to one or more standardized FAO commodity items; when a food group corresponded to multiple commodities, the recommended intake was allocated across commodities according to the country-specific FBS fractional composition within that group, ensuring that the sum of allocated commodity intakes matched the original group-level recommendation (mass-balance preservation). Mixed or composite foods were disaggregated into their primary ingredients using standard recipe compositions prior to mapping, thereby enabling alignment with raw-commodity emission factors. For nutrient variables with missing commodity-level values, we imputed nutrient contents using mean values from chemically similar foods within the same FAO sub-category to maintain internal consistency of the harmonized dataset. Subsequent calibration of dietary scenarios was undertaken to examine varying levels of animal protein intake while maintaining constant levels across other food groups. Analyses were conducted using a constant population in 2022 within each country. This approach isolates the effects of guideline adherence from confounding population growth dynamics (see Supplementary materials, page 6, Supplementary Figure S1).

### 2.3 Animal product optimization framework

A two-tier nutritional prioritization framework was developed to evaluate animal product reduction scenarios. The baseline scenarios encompassed the original species-specific FBDG composition (hereafter referred to as “Baseline FBDG”) and total protein scaled to the World Health Organization's (WHO) recommendations of 60 grams per day (MaxProt), which corresponds to ~0.83 g/kg body weight/day for adults. By constraining total energy intake to national FBDG levels and maintaining total protein within recommended caps, the optimized dietary patterns inherently align with the Acceptable Macronutrient Distribution Ranges (AMDR) specified in the respective national guidelines. Nutrient-optimized scenarios were developed with the objective of maximizing key animal-derived nutrients, namely, vitamin A/B12 (MaxVitamins) and EPA/DHA omega-3 fatty acids (MaxFattyAcids). Each nutrient scenario was assessed under both baseline protein assumptions, yielding six policy-relevant dietary patterns. Sustainability performance was benchmarked against EAT-Lancet minimum animal protein thresholds (Joint WHO/FAO/UNU Expert Consultation, 2007), thus enabling an evaluation of the trade-offs between nutrient provision, environmental impact, and guideline compliance.

To elucidate the contributions of protein, it is imperative to note the distinction between total protein, which encompasses all food sources (including animal, legume, grain, and nut sources), and animal protein, which is derived exclusively from meat, dairy, eggs, and fish. The optimization scenarios achieved a scaling of total protein to 60 g/day, with animal protein varying according to nutrient prioritization. The contributions of plant protein were automatically adjusted to address the nutrient gaps that resulted from reduced animal protein intake. Nutritional adequacy was assessed against reference values for protein, vitamin A, vitamin B12, and EPA/DHA, using multiple validation cohorts to ensure biological feasibility. It was acknowledged that there are differences in the quality of protein from plant and animal sources, but these differences were not explicitly modeled. This finding is consistent with the assumptions of the WHO regarding mixed diets.

The EAT-Lancet planetary health diet was used as a comprehensive reference framework to benchmark the environmental sustainability of dietary scenarios, establish minimum constraints for animal product intake and provide a comparative standard for evaluating optimized dietary outcomes. Daily ranges were established for red meat (0–28 g), poultry (0–58 g), fish (0–100 g), dairy (0–500 g) and eggs (0–25 g), ensuring essential nutrient provision while permitting country-specific optimization. Comparisons between optimized national diets and EAT-Lancet benchmarks highlight the potential for environmental improvement and reveal where cultural and nutritional considerations may constrain feasible dietary transitions.

### 2.4 Life cycle assessment

The environmental impacts of diets were assessed using a life cycle assessment (LCA) framework based on ISO 14,040/44 standards. The system boundary encompasses the entire cradle-to-farm process, including feed production, enteric fermentation, and manure management. National emission factors were derived from peer-reviewed studies and normalized using mass allocation and 20-year global warming potential (19, 28). This methodological choice ensures consistency while accounting for cross-national differences in production system intensities.

### 2.5 Nutritional contribution

This study evaluated protein, vitamin A, vitamin B12, and omega-3 fatty acids [α-linolenic acid (ALA), eicosapentaenoic acid (EPA), and docosahexaenoic acid (DHA)] in diets aligned with FBDG criteria. While ALA is widely available from plant sources, EPA and DHA served as the main indicators of long-chain omega-3 intake. Nutrient composition tables are provided (Supplementary materials, p.1). Optimization focused exclusively on animal protein, without modeling plant protein substitution, to examine the sustainability potential of animal-based dietary shifts. Total nutrient intake was calculated by combining food quantities with nutrient composition across scenarios (Supplementary materials, pp.3–4).

### 2.6 Economic costing framework

Instead of household affordability, this study adopted a production-side costing approach to evaluate the efficiency of agricultural resources across optimized dietary patterns. Producer price data were drawn from FAO Food Balance Sheets and national agricultural statistics, converted to constant 2020 international dollars using FAO purchasing power parity (PPP) factors. For marine products with incomplete domestic data, import valuations served as proxies. Prices were harmonized via consistent PPP adjustments to enable cross-country comparison.

This framework captures production-side resource intensity rather than consumer-facing costs, which are shaped by processing, distribution, retail markups, subsidies, and market structures. For instance, dairy may appear inexpensive at the producer level yet costly at retail, whereas direct-market vegetables show tighter price alignment.

PPP-adjusted producer prices were applied to normalize cross-country agricultural costs, enabling the assessment of dietary optimization from the perspective of production efficiency rather than consumer affordability.

Accordingly, results are interpreted as indicators of production efficiency and resource allocation, not affordability. By employing PPP-based producer prices, this study highlights the potential to interpret FBDG optimization through the lens of agricultural sustainability, thereby informing policy design and subsidy allocation (The accounting framework is shown in Supplementary Table S5).

### 2.7 Multi objective optimization framework and statistical Analysis

We used a multidimensional framework to evaluate the trade-offs between environmental impact, economic cost and nutritional adequacy across different dietary optimization scenarios. Rather than relying on a single algorithm, we systematically compared six predefined scenarios per country: two baseline scenarios (Original FBDG and MaxProt) and four nutrient-optimized scenarios targeting either vitamin A/B12 (MaxVitamins_FBDG and MaxVitamins_Protein) or EPA/DHA omega-3 (MaxFattyAcids_FBDG and MaxFattyAcids_Protein). This was done under both FBDG-native and WHO protein constraints. Each candidate solution represents one feasible ASF intake vector Qi generated under the scenario constraints (i.e., one point in 8). Cross-country relationships between objectives were quantified using Pearson correlation coefficients, and for we additionally fitted ordinary least squares (OLS) linear regressions of dietary cost against environmental impact across candidate solutions within each country; the reported R2 values correspond to these regressions. For each OLS model, we report the slope estimate with 95% confidence intervals and two-sided *P*-values to characterize statistical uncertainty. For each scenario, three key performance indicators are calculated:

Economic cost: Total daily dietary cost via FAO PPP-adjusted producer prices:


$$\begin{array}{l} C\left(x\right)=\underset{i}{\sum }Q_{i}\times R_{i} \end{array}$$

Environmental impact: Life-cycle greenhouse gas emissions:


$$\begin{array}{l} E\left(x\right)=\underset{i}{\sum }\left(Q_{i}\times EF_{i}\right) \end{array}$$

Subject to nutritional constraints:


$$\begin{array}{l} N=\left(\underset{i}{\sum }\left(Q_{i}\times N_{j}\right)\right)/RDA_{j}\mathrm{for}\;\mathrm{each}\;\mathrm{nutrient}\text{ j} \end{array}$$

Here, *Q*_*i*_ denotes the daily consumption of food category *i, P*_*i*_ is the PPP-adjusted cost coefficient, *EF*_*i*_ is the environmental footprint coefficient, *N*_*ij*_ is the nutrient content, and *RDAj* is the recommended daily allowance. Nutritional constraints were enforced via an adaptive penalty function, applying quadratic penalties proportional to the severity of constraint violations. Specifically, violations were defined as $d_{j}=\max\left(0,RDA_{j}-\underset{i}{\sum }Q_{i}N_{ij}\right)$ and penalties scaled with ∑_j_d2 j to prioritize feasible solutions.

Scenario performance was visualized via boxplots, heatmaps, and radar charts. The environmental benchmarks were informed by reductions relative to baseline FBDGs, economic feasibility by relative cost changes, and nutritional adequacy by maintaining the essential nutrient supply above minimum thresholds. Unlike weighted-sum approaches that assign arbitrary coefficients to conflicting goals, the Non-dominated Sorting Genetic Algorithm II (NSGA-II) algorithm treats nutrition, environment, and cost as simultaneous, equal-priority objectives (29).


$$\begin{array}{l} Pareto\;Optimality:\\ ∄x^{\prime }\in X\;such\;that\;f_{i}\left(x^{\prime }\right)\leq f_{i}\left(x\right)\forall i\;and\;f_{j}\left(x^{\prime }\right)<f_{j}\left(x\right)\;for\;some\;j \end{array}$$

**Figure 1:**
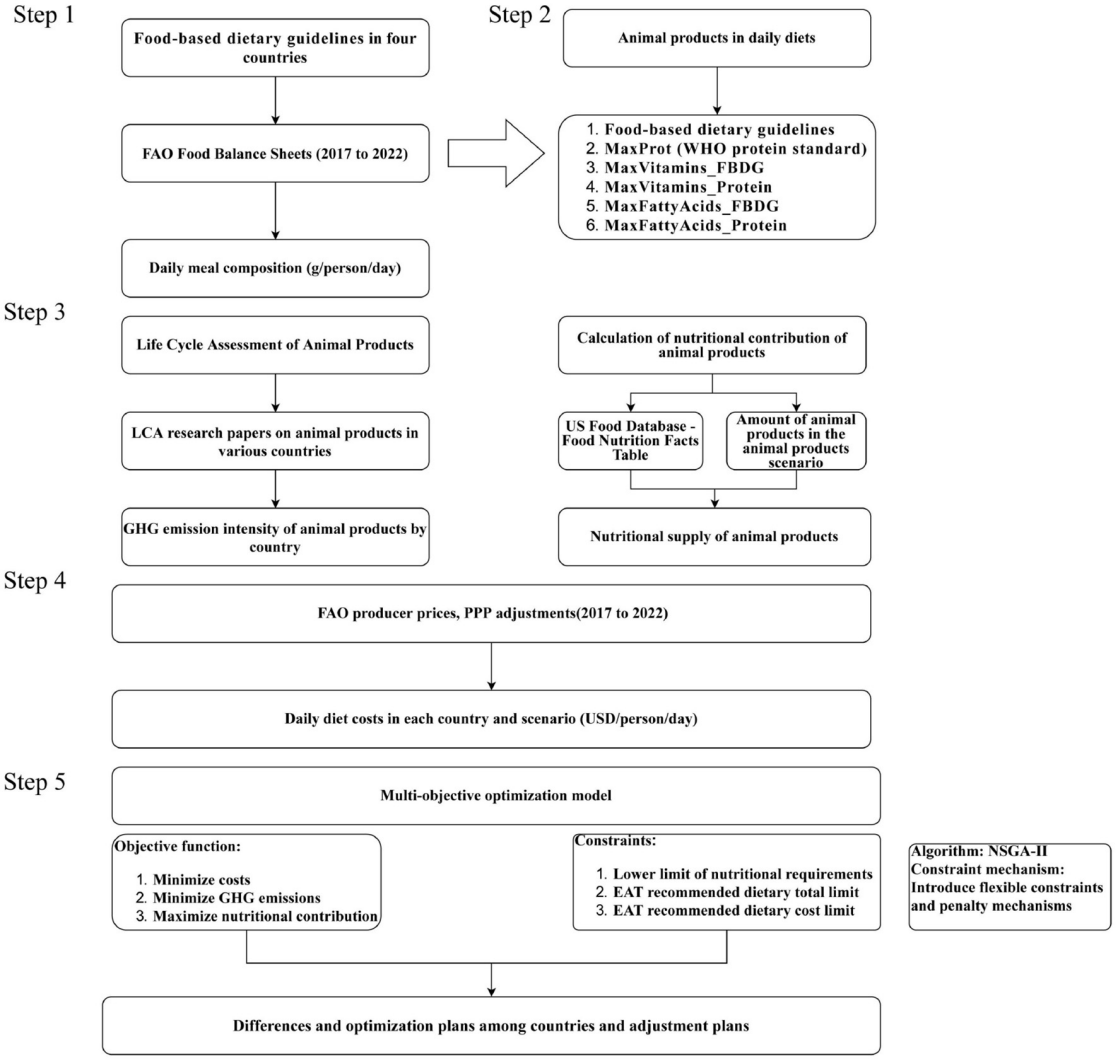
Flowchart of the method. The model and data input process are briefly described.

This approach generates a non-dominated solution set, known as the Pareto frontier. The Pareto frontier represents the set of optimal solutions where it is impossible to improve one objective (e.g., reducing emissions) without compromising another (e.g., increasing cost or reducing nutrient density). This methodology allows us to visualize the efficiency limits of different dietary trade-offs without the bias of pre-assigned weighting coefficients. Sensitivity analyses tested robustness to variations in key parameters, including price volatility and constraint thresholds. The analysis revealed that the Pareto-optimal solutions maintained structural stability, exhibiting low sensitivity (< 5% variation) to greenhouse gas outcomes (25). This framework enables the identification of dietary scenarios that balance sustainability, economic viability, and nutritional integrity, supporting evidence-based policy for context-specific sustainable diet transitions (Figure 1).

### 2.8 Uncertainty and sensitivity analysis

To evaluate the robustness of the optimization framework, we conducted Monte Carlo simulations and systematic sensitivity analyses. The NSGA-II hyperparameters were configured to balance exploration and exploitation: population size of 200, 100 generations, crossover probability of 0.7, and mutation probability of 0.2.

We modeled uncertainty through multiplicative perturbations applied to baseline parameter values. The perturbed parameter value p' was calculated according to the relation:


$$\begin{array}{l} p^{\prime }=p\times \left(1+\varepsilon \right) \end{array}$$

where *p* represents the baseline parameter value and ε denotes a random perturbation drawn from a normal distribution with zero mean and variance σ^2^, expressed as ε ~ *N*(0, σ^2^). The standard deviation was set at σ = 0.10, representing ten percent baseline uncertainty in model parameters. This multiplicative formulation appropriately captures the compounding nature of real-world uncertainties inherent in price volatility and resource availability parameters, as such factors typically scale proportionally rather than additively with parameter magnitude. We performed 500 independent Monte Carlo simulation runs, each initialized with a distinct random seed to ensure statistical independence across realizations. This sample size was deemed sufficient based on convergence validation criteria described below.

Systematic sensitivity analysis was conducted by applying deterministic perturbations to key input parameters at five levels: 5%, 10%, 15%, 20%, and 25% relative to baseline values. This graduated approach allowed us to characterize the linearity or nonlinearity of the system response across a practical range of input variations. Particular attention was directed toward price volatility and fish availability, as these parameters represent the most significant sources of real-world variability.

All results are reported as sample means accompanied by standard deviations and 95% confidence intervals Robustness was summarized using the coefficient of variation *CV* = *SD*/*mean* for the environmental impact metric across Monte Carlo realizations. Calculated using the standard normal approximation. Convergence adequacy was verified by comparing key statistical measures at sample sizes of 500, 1,000, and 2,000 Monte Carlo runs. Convergence was deemed acceptable when differences in mean values remained below 1 percent, confirming that 500 runs provided sufficient statistical precision for our analysis.

## 3 Results

### 3.1 Current dietary guideline assessment and optimization opportunities

The selected countries are illustrative of key developmental archetypes within the global food system. The United States and Australia are representative of high-income economies, with well-established livestock industries and high per capita animal product consumption patterns. The People's Republic of China is a pertinent case study for the analysis of the dynamics of a rapidly developing economy experiencing significant dietary transition. This is characterized by an increase in the consumption of animal products alongside persistent nutritional disparities. New Zealand offers invaluable insights from an export-oriented agricultural economy, where domestic dietary patterns intersect with global food trade dynamics (Table 1).

**Table 1:** Key characteristics of food production systems and dietary guidelines in selected countries.

**Characteristic**	**United States**	**China**	**Australia**	**New Zealand**
Economic archetype	High-Income	Developing/Transition	High-Income (Export)	High-Income (Export)
Dominant livestock	Beef, Poultry, Dairy	Pork, Poultry, Aquaculture	Beef, Sheep	Dairy, Beef, Sheep
Net trade status	Net Exporter	Net Importer (Feed/Meat)	Major Net Exporter	Major Net Exporter
Primary FBDG	Dietary Guidelines for Americans (MyPlate)	Dietary Guidelines for Chinese Residents (Pagoda)	Australian Dietary Guidelines	Eating and Activity Guidelines

**Figure 2:**
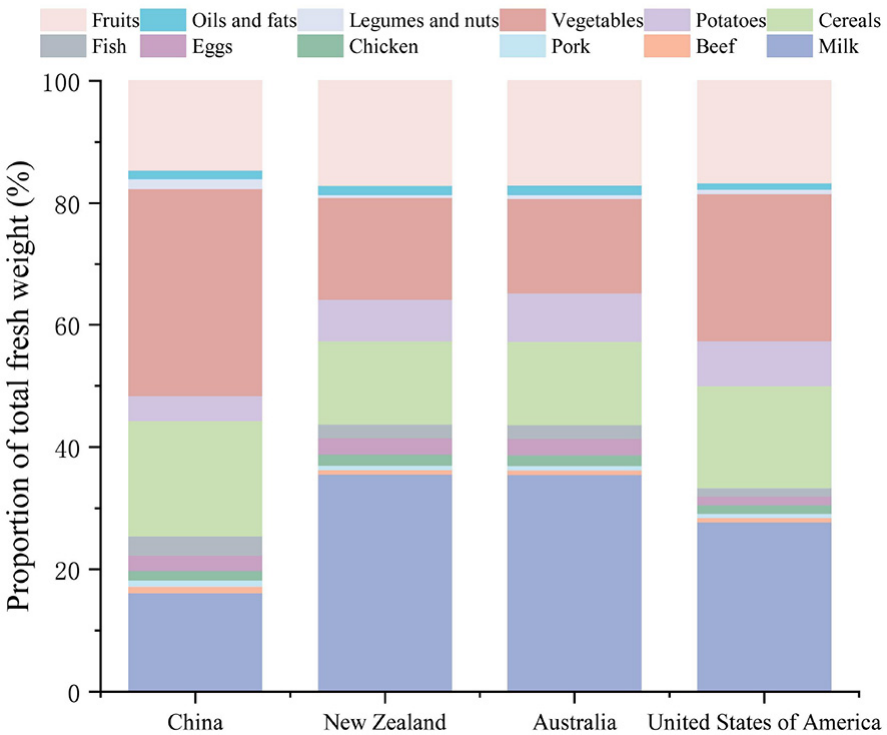
Composition of raw animal products from selected countries. Composition of animal products in the original FBDGs (grams per capita per day). Data derived from national dietary guidelines (Supplementary Table S1).

An analysis of national dietary guidelines in Australia, New Zealand, the United States and China revealed systematic overconsumption and heterogeneity in the composition of animal proteins (see Figure 2). Australia and New Zealand displayed similar profiles, emphasizing ruminant products due to shared geographical and industrial factors, indicating the potential for policy coordination. In contrast, the United States prioritized beef and poultry (53% of total protein from animal sources), whereas China focused on pork (41% of animal protein).

All countries exceeded health and environmental benchmarks, with recommended protein intakes 25–47% above WHO guidelines (30, 31) and with animal proteins accounting for 41%−53% of total intake vs. the optimal level of 25%−35%. It is worth noting that the regional environmental emission factors calculated in this study are 18–32% higher than the FAO industry average. This is attributed to our inclusion of indirect land-use change and supply chain inputs in the calculation. The United States presented the greatest excess (Table 2; 85 g total protein, of which 45 g was animal-derived; 42% above target), followed by Australia and New Zealand (Table 2; 85–88 g total protein, of which 43 g was animal-derived), and China (Table 2; 87 g total protein, of which 36 g was animal-derived).

**Table 2:** Protein contribution of FBDGs in four countries per capita per day, by population average.

**Country**	**Total protein contribution (FBDG)**	**Animal protein contribution (FBDG)**	**Share animal protein/total protein FBDG**
Australia	85 g	43 g	0.51
China	87 g	36 g	0.41
New Zealand	88 g	43 g	0.49
The United States	85 g	45 g	0.53

FBDG = Food-Based Dietary Guidelines. All values represent recommended daily intakes for adults (g/day) based on national guidelines current at the time of analysis. Values in parentheses indicate standard errors where data sources provided variance estimates.

**Figure 3:**
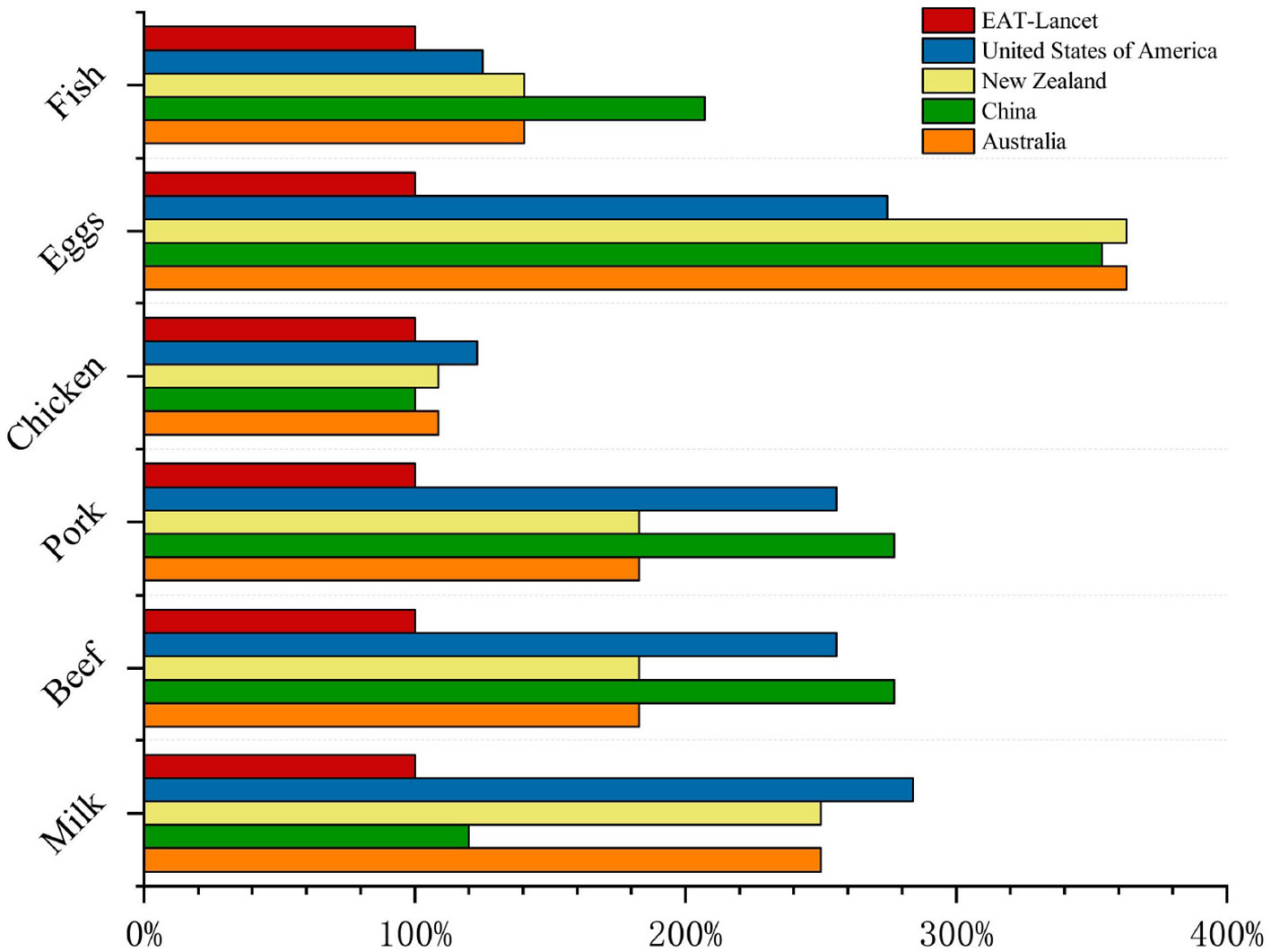
Differences between animal products in the original dietary guidelines and those recommended by the EAT-Lancet Commission. “100%” is equal to the daily intake recommended in the EAT-Lancet dietary guidelines, which includes 28 grams of fish, 7 grams of pork, 13 grams of eggs, 29 grams of poultry, 7 grams of beef and 250 grams of milk. (See Supplementary Table S6 for specific value ranges.). All units are in grams per person per day (g/capita/day). Dotted lines represent the EAT-Lancet reference targets.

Eggs were the most overconsumed category, averaging 2.4 times the EAT-Lancet recommendation, with excesses of 3.1 and 2.8 times in Australia and New Zealand, respectively. These findings highlight the potential for reallocating animal products in a targeted manner to improve health and environmental outcomes while maintaining cultural acceptability (see Figure 3).

### 3.2 Optimization scenario performance analysis

**Figure 4:**
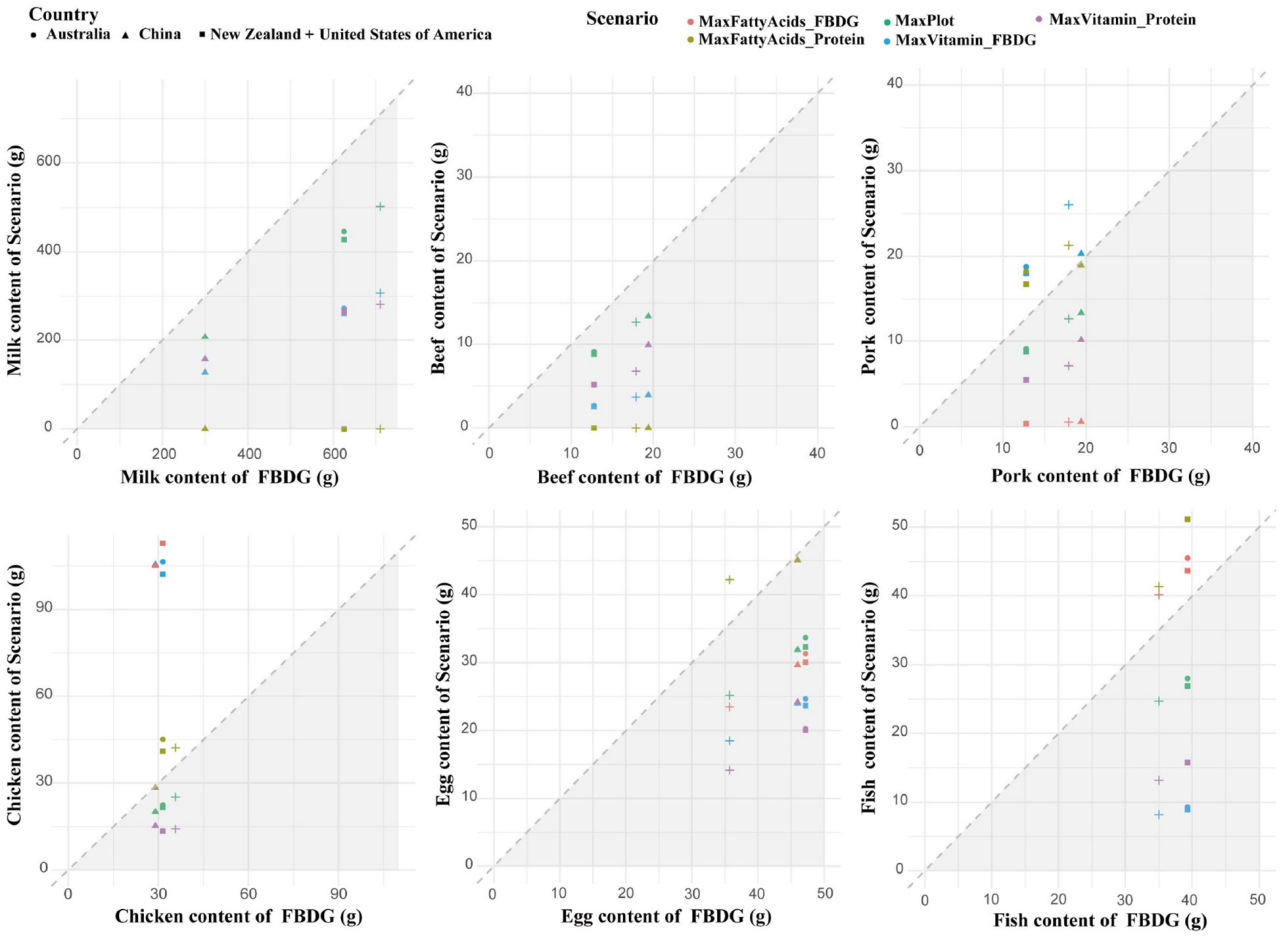
Changes in animal products compared with the original FBDGs under multiple scenarios. Comparison of the inclusion of animal products in the original dietary guidelines and the average daily proportions of animal products in different scenarios. The diagonal lines indicate that the nutrient contributions in the scenarios and the dietary guidelines are equal. For the values in the upper triangle, the food contribution in each scenario exceeds the dietary guidelines, whereas in the lower triangle (gray shading), the food contribution in each scenario is below the dietary guidelines. The horizontal differences represent the differences between countries, and the vertical differences represent the differences between scenarios. FBDG = food-based dietary guideline. MaxProt= Scale protein based on the WHO recommendation of 60 g protein per person per day. MaxVitamins_FBDG = maximizing vitamins based on the FBDG context. MaxVitamins _Protein = maximizing vitamins on the basis of the WHO context. MaxFattyAcids _FBDG = maximized fatty acid scenario in the FBDG context. MaxFattyAcids _Protein = maximized fatty acid scenario in the WHO context.

Fatty acid optimization scenarios showed that modifying animal product intake alone could not supply adequate EPA and DHA, underscoring reliance on marine or fortified sources. Regulate ruminant products (beef, milk) consistently shifted intake toward poultry and fish, with chicken as the dominant substitute across all countries (see Figure 4). Protein-constrained diets drove further increases in fish consumption, except in China where levels were already high.

Vitamin-focused optimization within existing FBDGs elevated pork and chicken intake but reduced other animal products by ~50%. Under protein restrictions, animal products declined across all nations, though reductions in fish, milk, and beef were more moderate.

Gray shaded regions in Figure 4 highlight nutritional shortfalls, illustrating trade-offs where prioritizing single nutrients disrupts overall dietary balance. These results indicate that, within current production limits, animal-source foods (ASF) alone cannot simultaneously satisfy nutritional adequacy and environmental targets. Nevertheless, their role remains critical, reinforcing the need for holistic, multi-nutrient optimization rather than simple substitution. This optimization is biologically supported by emerging ruminant microbiome research. Studies demonstrate that ruminal metabolic redundancy buffers community changes during feed transitions, maintaining nitrogen cycling efficiency (32), This physiological resilience ensures that ruminant production remains efficient even under environmentally driven management shifts, thereby supporting dietary strategies that balance moderate animal-source foods (ASF) intake with emission reductions.

### 3.3 Nutrient requirements for each treatment under the different scenarios

**Figure 5:**
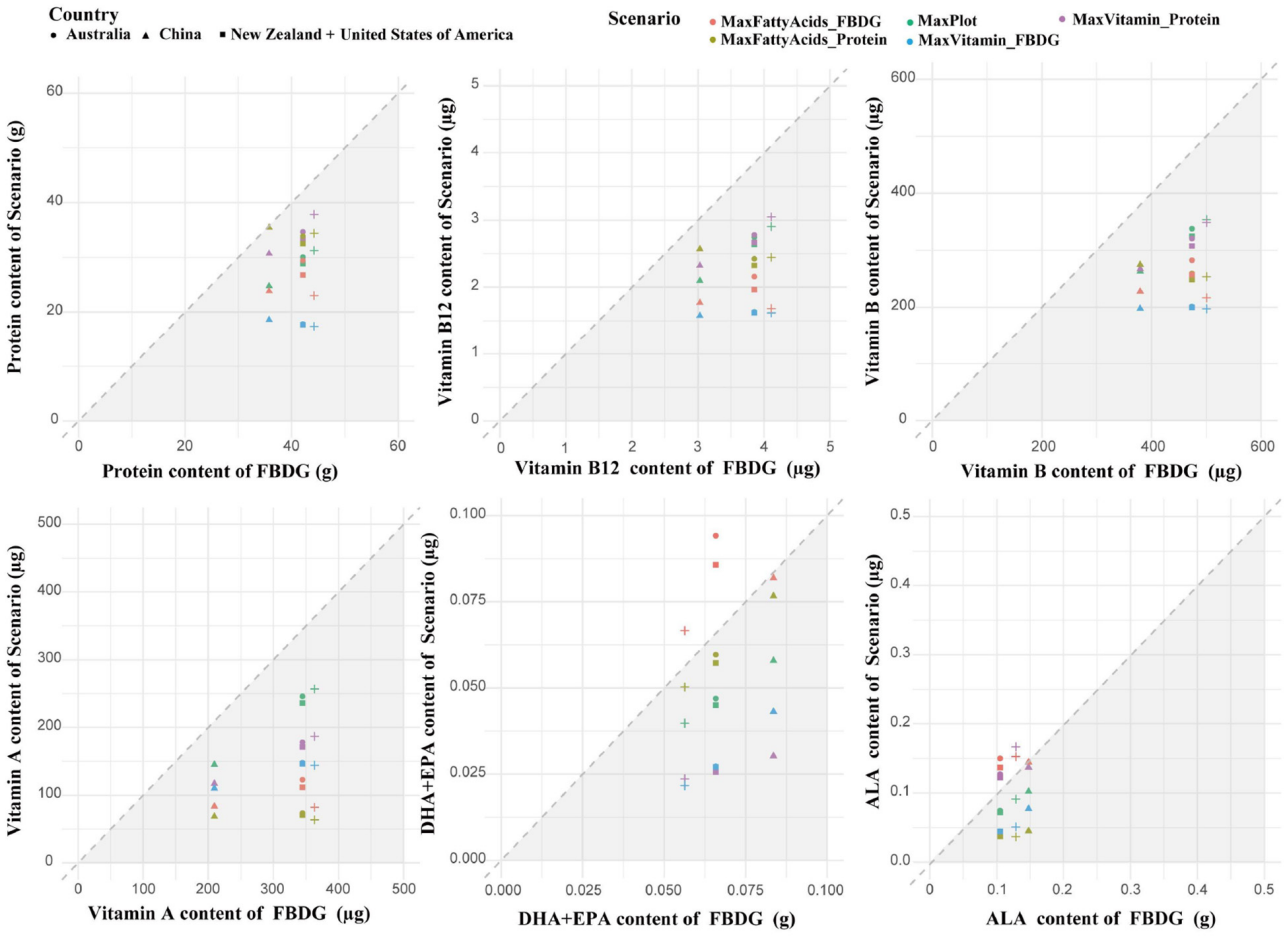
Changes in nutrients provided by animal products compared with those provided by original FBDGs under multiple scenarios. Nutrients of the animal products in the original food-based dietary guidelines vs. nutrients of the animal products in the scenarios and countries per day per capita. The diagonal line indicates equal nutritional contributions in the scenarios and dietary guidelines. For values in the upper triangle, the nutrient contributions of the scenarios exceed those of the dietary guidelines, and in the lower triangle (gray shading), the nutrient contributions of the scenarios are lower than those of the dietary guidelines. The horizontal differences indicate differences between countries, and the vertical differences indicate differences between scenarios. ALA = α-linolenic acid. DHA = docosahexaenoic acid. EPA = eicosapentaenoic acid.

A multi scenario analysis was conducted to identify key nutrient trade-offs. This analysis demonstrated an inverse relationship between DHA+EPA levels and the adequacy of vitamins A and B12 (see Figure 5). Notable national variations emerged: Despite a higher baseline of DHA+EPA from fish in its native FBDGs, China experienced a 38% reduction under the WHO protein limit of 60 g/day, dropping below the levels reported in the United States and Australia. Oceania (Australia and New Zealand) exhibited significant vitamin–fatty acid trade-offs because of its dairy-centric dietary patterns.

In unconstrained scenarios, protein and fatty acids exhibited synergistic effects. However, a 10% increase in DHA+EPA was linked to a 15–22% decrease in vitamin A. In protein-restricted scenarios, animal protein intake varied: 24.8 g/day in China and Australia, compared with 31.3 g/day in the United States. Adaptations were tailored to each country. China has reduced beef consumption while increasing fish consumption, the United States has shifted toward poultry consumption, New Zealand has favored pork consumption over egg consumption, and Australia has balanced beef and milk consumption.

Optimized frontier analysis (shaded area in Figure 5) identified policy thresholds: developed nations require sustained beef and milk consumption to meet the EAT-Lancet recommendations for DHA and EPA, whereas China should increase aquaculture production to address protein shortages. These findings advocate nutrient-specific prioritization in FBDGs rather than reducing the number of animal products indiscriminately. Integrated plant–animal approaches incorporating legumes or fortified foods could reduce GHG emissions and costs by 10–20%. Cell-based meats could provide an equivalent nutrient profile with reduced environmental impact in high-income settings (33, 34).

### 3.4 Greenhouse gas emissions and cost burdens across countries and scenarios

**Figure 6:**
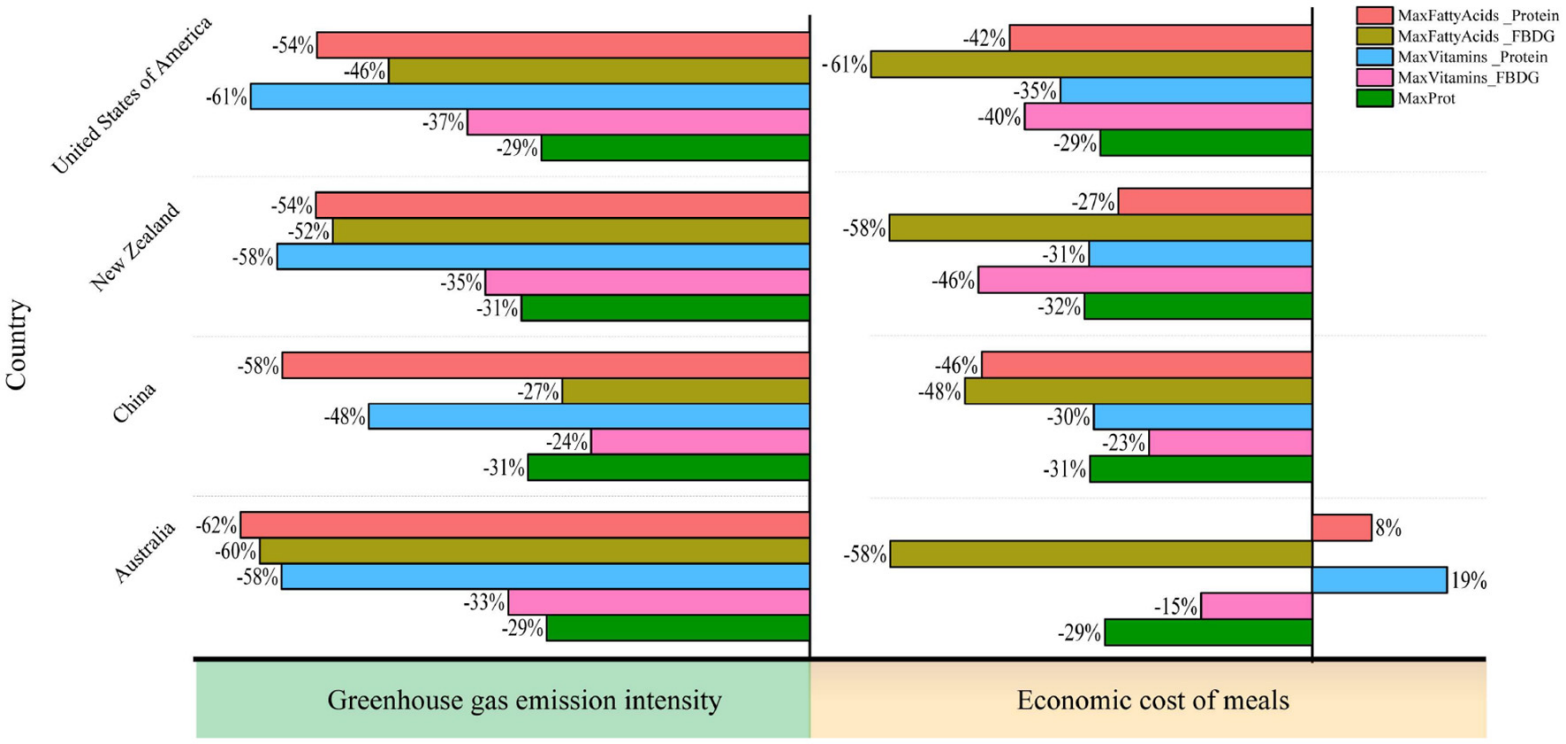
Changes in the environment and economy compared with those of the original FBDGs under multiple scenarios. Changes in greenhouse gas emission intensity and dietary economic costs of animal products compared with the original dietary guidelines under each scenario. The left side of the figure shows the comparison of greenhouse gas emission intensity of animal products in various adjustment plans of various countries and the original dietary guidelines, and the right side shows the changes in per capita dietary burden of various adjustment plans of various countries compared with the original dietary guidelines.

The complete elimination of animal products is impractical, as this conflicts with global dietary guidelines that promote balanced diets. Optimized adjustments must consider national production conditions. While all five scenarios reduced greenhouse gas emissions, the economic impact varied (see Figure 6).

The MaxProt scenario, which involves making minimal adjustments to animal components, maintains nutritional levels close to the FBDGs. Emission reductions ranged from 29% (AU/US) to 31% (NZ/CN), while cost reductions averaged 12.3%, with the largest decreases in NZ (-32%) and the US/AU (-29%).

The FBDG-based nutrient optimization scenarios presented divergent patterns. In the ‘MaxVitamins' scenario, GHG reductions ranged from−27% in China to−37% in the US., with economic costs decreasing in China (-23%) but increasing in Australia (+19%). The MaxFattyAcids scenario yielded the largest emission reductions (an average of−46.2%), with Australia achieving a reduction of−60%, accompanied by substantial cost reductions (-60%).

Under WHO protein constraints, the MaxVitamins scenario led to reductions in GHG emissions of 48–61%, with cost changes ranging from +15% in Australia to−35% in the US (average−22.6%). The MaxFatty Acids-WHO achieved emission reductions of 54–62% and average cost decreases of 33.4%, demonstrating the synergy between fatty acid-focused optimization and economic feasibility.

Overall, scenarios that achieved greater emission reductions were often associated with lower economic costs, whereas those that involved moderate adjustments exhibited less consistent patterns. These findings emphasize the importance of context-specific trade-offs between environmental impact, nutrient adequacy and economic burden and highlight the need for tailored national strategies to ensure sustainable dietary transitions.

### 3.5 Reconciling nutrition, cost, and environmental goals: sustainable dietary transition pathways in four countries

**Figure 7:**
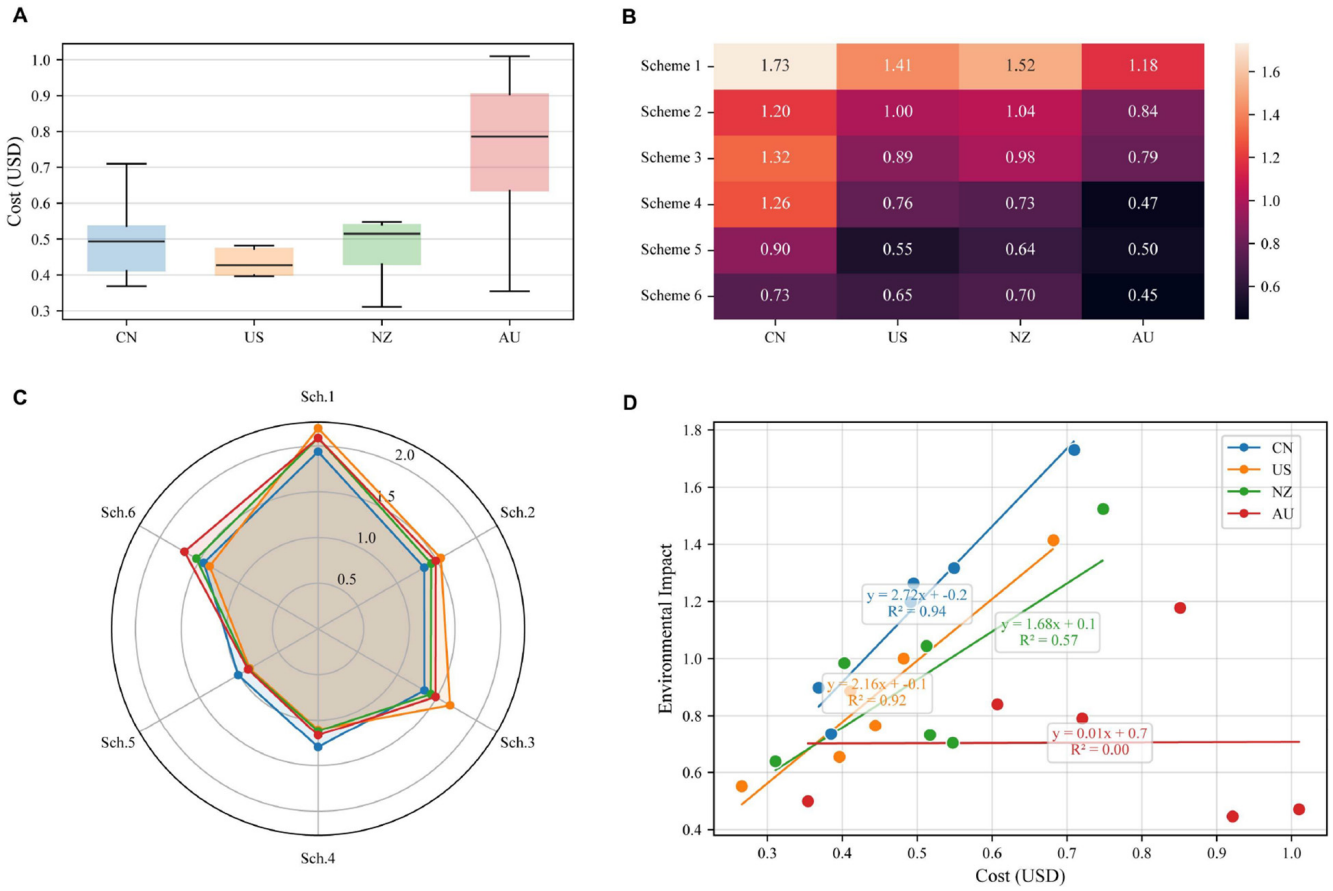
Environmental, economic and nutritional subindicators for each option. **(A)** shows the average dietary cost of each country, and **(B)** shows environmental impact heatmap illustrating the greenhouse gas emission intensity of each dietary scenario relative to the baseline. Scenarios (Schemes 1–6) correspond to: 1) Baseline FBDG, 2) MaxProt (Protein-constrained), 3) MaxVitamins (FBDG-constrained), 4) MaxVitamins (Protein-constrained), 5) MaxFattyAcids (FBDG-constrained), and 6) MaxFattyAcids (Protein-constrained). Darker colors indicate higher relative environmental intensity. **(C)** is a nutritional radar chart of each dietary plan in each country, which determines the nutritional supply of each plan in each country on the basis of the nutritional contribution of animal products recommended by the EAT. **(D)** is the cost–environment regression fit of each dietary plan in each country. The linear equation is constructed on the basis of the nutritional contribution and cost burden of animal products in each country to determine the internal connection between the nutritional environment impact and cost burden of each country.

A multidimensional analysis quantified trade-offs among dietary cost, nutritional adequacy, and environmental impact across four countries (Figure 7). Cost distributions differed markedly: Australia exhibited the highest costs and the United States the lowest, with pronounced outliers in several scenarios (see Figure 7).

Linear regression of dietary cost against environmental impact across candidate solutions indicated strong associations in China *R*^2^ = 0.94 and the United States *R*^2^ = 0.92, whereas New Zealand showed a weaker relationship *R*^2^ = 0.325 (Figure 7B). In Australia, low-impact solutions were predominantly located in a higher-cost region of the solution space (Figure 7B).

Scenario-level comparisons further indicated that nutrient-prioritized settings were associated with higher environmental impacts in China relative to baseline settings, while Australia and New Zealand—despite comparable animal-product proportions—showed divergent cost and environmental outcomes (Figures 7C–D).

**Figure 8:**
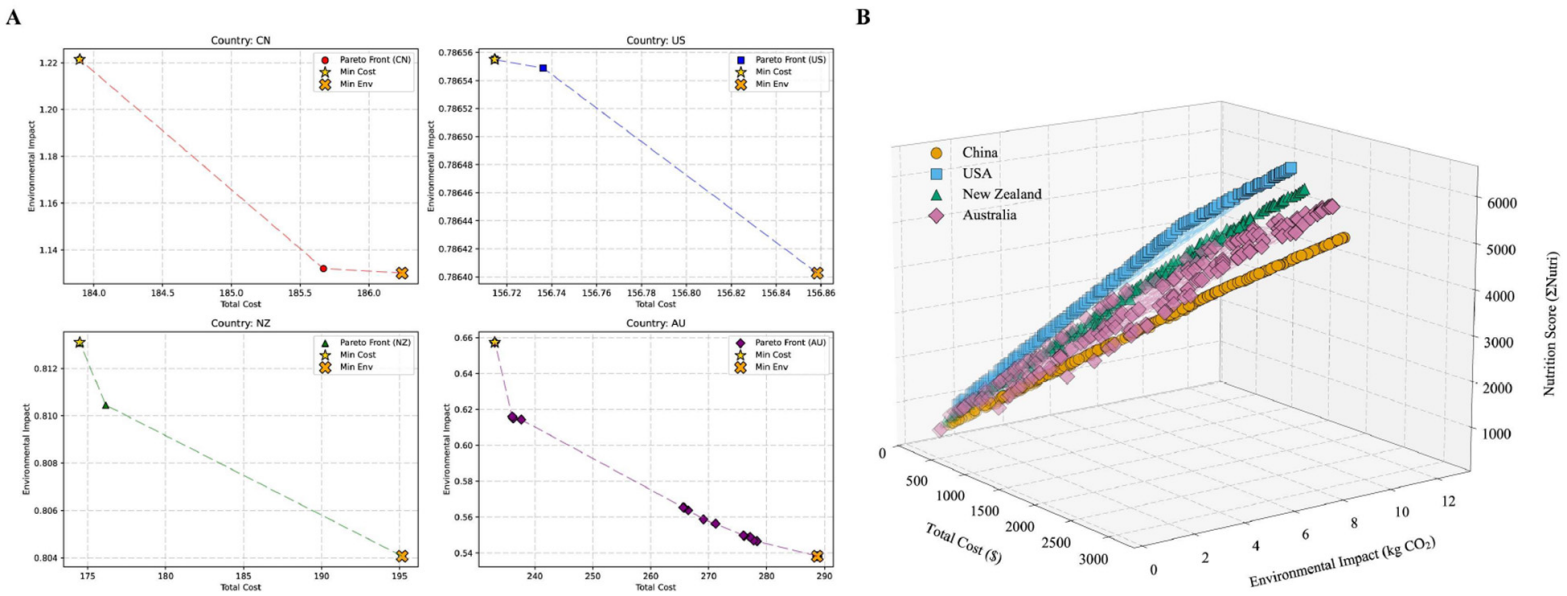
Pareto frontiers of dual and triple objectives for different countries. **(A)** Country-specific Pareto frontiers illustrating the trade-offs between economic cost and environmental impact. Note: Individual axes are used to preserve the legibility of national trajectories given divergent absolute scales. **(B)** Global comparative landscape showing all optimized solutions for China, USA, Australia, and New Zealand within a unified 3D coordinate system (Cost, Environment, and Nutrition). This allows for a direct comparison of the feasible solution space across different national contexts.

Multi-objective genetic algorithm optimisation delineated country-specific Pareto frontiers (see Figure 8). China's Pareto-optimal solutions tended toward lower emissions at higher costs; the United States achieved lower costs at modest environmental impacts; and Australia's frontier reflected higher costs under nutrition-prioritized solutions (35).

### 3.6 Uncertainty and sensitivity analysis of the optimization solutions

Monte Carlo simulations revealed substantial heterogeneity in optimization stability across countries (see Supplementary Figure S2A). The United States exhibited the greatest robustness in cost optimization, with a coefficient of variation of 0.053, whereas China showed the greatest sensitivity, with a coefficient of variation of 0.071. This reflects vulnerabilities in emerging, trade-dependent food systems. Australia's outcomes were highly concentrated, achieving optimal performance under environmental minimization scenarios.

Country-specific sensitivity profiles varied by animal-based food category. In China, eggs displayed the highest elasticity (0.37 at a 5% perturbation), whereas pork remained structurally stable under larger perturbations (15–20%). In the US, beef exhibited increased sensitivity (~0.20 under high perturbation), whereas fish sensitivity remained consistent across scenarios. This highlights the importance of strategies that diversify protein sources. Pork was New Zealand's most sensitive category (elasticity 0.51 at 5% perturbation), whereas Australia showed the highest overall sensitivity to pork price fluctuations (elasticity 0.70 at 5%), indicating critical dependence on market stability.

Overall, the heterogeneity underscores the need for country-specific implementation strategies. Highly sensitive systems (e.g., China and Australia) may require conservative rollouts and market stabilization policies, whereas more stable systems (e.g., the US) afford greater flexibility in timing and intervention design. Future models that integrate temporal, demographic and health-related constraints will enhance the relevance of policies for sustainable dietary transitions.

## 4 Discussion

Food-based dietary guidelines (FBDGs) have historically focused on nutritional adequacy and chronic disease prevention, with environmental sustainability considered only incidentally. Epidemiological evidence consistently shows that dietary patterns emphasizing plant foods with moderate animal product intake—such as Mediterranean and DASH diets — are associated with reduced risks of cardiovascular disease, type 2 diabetes, and certain cancers (36–38). Our optimization scenarios achieve 28–62% reductions in animal products while maintaining essential nutrient adequacy.

### 4.1 Balancing nutritional adequacy with environmental limits

These health-optimal patterns preserve 28%−36% of protein intake from animal sources to meet the requirements for vitamin B12, and preformed vitamin A (39). This underscores the indispensable role of animal-source foods in supplying certain bioavailable micronutrients that are difficult to obtain from plant-based sources alone (40, 41). Their strategic inclusion, even at reduced levels, is therefore essential to balance nutritional adequacy with environmental sustainability in dietary transition pathways.

Analysis of FBDGs in Australia, the United States, China, and New Zealand revealed systematic overconsumption of animal products, exceeding global health recommendations and EAT-Lancet planetary boundaries by 22%−38% (Figure 2). Crucially, these observed overconsumption patterns reflect excess total caloric intake, not merely animal product consumption. However, animal-source foods (ASF) are disproportionately implicated due to their high environmental footprint per calorie. Our optimization approach addresses this by constraining total energy intake to recommended FBDG levels (isocaloric targets) while optimizing animal product composition. Eggs and chickens are consistently overrepresented, with eggs 2.4 times more common on average and up to 3.1 times more common in Australia (Figure 3). Targeted reductions of 29%−32% in animal protein can achieve substantial GHG reductions while maintaining nutritional adequacy, although specific micronutrients (vitamins A and B12) may require plant-based fortification (42). It is also important to interpret our use of the EAT-Lancet reference framework with nuance. While we utilized EAT-Lancet targets to benchmark environmental sustainability, we acknowledge recent critiques suggesting that the Commission's strict limits on red meat may not universally align with mortality data from the Global Burden of Disease study (43). Our optimization results support this perspective: we found that strict adherence to minimum EAT-Lancet values could compromise micronutrient adequacy (specifically Vitamin B12 and A). Therefore, our model prioritizes maintaining these essential nutrients, resulting in optimized diets that retain moderate amounts of animal-source foods—often exceeding the theoretical minimums—to ensure health outcomes are not sacrificed for environmental goals.

We also acknowledge the critical issue of nutrient bioavailability. A complete shift to plant-based diets can introduce risks of micronutrient deficiencies due to lower bioavailability and the presence of anti-nutrients. Our results demonstrate that retaining moderate amounts of animal-source foods (29–62% reduction rather than 100%) is often necessary to meet these nutrient requirements cost-effectively, particularly for vulnerable populations where fortified plant-based alternatives may be less accessible.

### 4.2 Quantifying mitigation potential and economic feasibility

Our analysis quantifies the ‘quantum of unsustainability' driven by current overconsumption. By aligning consumption patterns closer to the optimized targets—which effectively cap animal product intake at levels comparable to EAT-Lancet recommendations plus a tolerance margin—we demonstrate that national GHG emissions from the food sector can be reduced by 25–60% without compromising nutritional adequacy. This explicitly accounts for the environmental burden that could be circumvented by lowering consumption from current excessive levels to nutritionally adequate baselines.

Crucially, the environmental mitigation potential identified in this study is underpinned by a dual mechanism: the substitution of high-emission protein sources and the systematic correction of total caloric intake. Current baseline diets in the studied high-income nations are characterized by significant caloric overconsumption. In our modeling framework, all optimized scenarios were strictly constrained to meet national FBDG energy recommendations (isocaloric targets), effectively simulating a transition where population-level intake is moderated to nutritionally adequate levels. Consequently, the projected 25–60% reductions in GHG emissions reflect the combined impact of structural dietary shifts and the elimination of the environmental burden associated with excess caloric consumption.

However, these analyses did not incorporate temporal dynamics, such as seasonal production variability or demographic shifts, which limits direct extrapolation to real-world contexts. Nevertheless, the findings corroborate earlier results regarding the optimal allocation of animal products, particularly the role of eggs in China and the US.

Furthermore, our approach is designed to prioritize national feasibility rather than imposing a uniform global prescription. Culturally, instead of optimizing toward a global reference diet that may require substantial changes in food preferences and culinary practices, we treat each country's existing food-based dietary guidelines (FBDGs) as the baseline constraint set. The optimization therefore reallocates the relative shares of food groups already recognized in national guidance, which reduces the extent of behavioral departure and supports interpretability and policy relevance.

Economically, we use PPP-adjusted producer prices to characterize the production-side cost structure underlying dietary transitions. We acknowledge that retail prices are more directly related to consumer affordability; however, they incorporate additional downstream components (e.g., retail margins, taxes, marketing, and distribution) that vary widely across countries and may confound cross-national comparisons of the underlying resource cost of producing foods. Producer prices more directly reflect farm-gate production costs and comparative advantages within national agricultural systems. PPP adjustment is applied to place producer prices on a comparable purchasing-power basis across countries. Importantly, our objective is not to infer household affordability, but to identify dietary shifts that are consistent with domestic production capabilities and structural resource costs. Consumer-facing affordability implications are therefore treated as complementary and would require retail price or household expenditure data, which are not consistently available across countries.

### 4.3 National heterogeneity and policy implications

Cross-country variation in optimized dietary scenarios reflects the distinct characteristics of existing dietary patterns and production systems (44–47). Under WHO protein constraints, models for China favored maintaining fish intake while reducing beef, whereas the US. trajectory shifted toward poultry, and Australia and New Zealand adopted distinct balances of beef, milk, and pork (Figures 4, 5). These national differences underscore that optimal dietary transitions are tethered to the heterogeneity of livestock production systems, particularly available feed resources. Comparative studies on diverse roughage sources demonstrate that local feed composition drives specific ruminal fermentation patterns and microbial community structures, which in turn dictate nutrient output efficiency (48, 49). These production-side biological factors effectively constrain the feasible space for dietary optimization, necessitating the development of country-specific intervention strategies. Fatty acid optimization increased fish and poultry consumption, whereas vitamin-targeted scenarios emphasized pork and chicken, illustrating nutrient-specific trade-offs. Reductions in ruminant products yielded 28–62% GHG reductions, with economic impacts ranging from −40% to +19% across countries (Figures 6–8), highlighting environmental–cost trade-offs.

Uncertainty and sensitivity analyses underscore the need for tailored policies. The US showed robust cost–environment correlations (R^2^ = 0.92), suggesting that carbon pricing and subsidies for low-emission protein could incentivize dietary shifts (50) China exhibited greater sensitivity to parameter perturbations, necessitating resilient supply chains and strategic aquaculture expansion (Supplementary Figures S2–S3). Australia and New Zealand, with export-oriented livestock systems, require interventions to reduce emission intensity while preserving competitiveness, including precision agriculture, feed management, and consumption-based carbon accounting (51–53).

The analysis yields four primary policy recommendations. First, governments should reallocate agricultural subsidies toward sustainable poultry and aquaculture production systems (54), as these sectors consistently yield optimal environmental and economic outcomes across modeled scenarios. However, distinct from simple species substitution, a second recommendation emphasizes the modernization of ruminant production, but with a critical caveat regarding ‘greenwashing'. Technological interventions, such as precision feeding strategies targeting rumen microbiome optimization, should not be used to justify ‘business-as-usual' expansion of industrial livestock systems. Instead, they offer a complementary mitigation pathway strictly for the reduced quantity of animal products that remain in a sustainable diet. Research demonstrates that enhancing nitrogen utilization efficiency through microbial management can significantly reduce environmental footprints without compromising productivity (55). Integrating these production-side biotechnological innovations with consumption-side dietary optimization creates a synergistic effect, amplifying emission reductions beyond the limits of dietary shifts alone. Second, the integration of fortified plant-based proteins addresses micronutrient gaps identified during dietary transitions while supporting emission reduction objectives (56). Third, targeted nutrition education programs require country-specific designs to accommodate cultural preferences and production systems, given the substantial cross-national variation in optimization outcomes (57). Fourth, market stabilization frameworks must monitor price volatility for sensitive commodities, particularly pork and eggs, which present high elasticity coefficients across perturbation scenarios (Supplementary Figure S2). International coordination mechanisms are essential for preventing emission displacement through trade channels (58). Unilateral dietary policies risk shifting environmental burdens to regions with less stringent regulations, necessitating coordinated frameworks that align national transition strategies with global sustainability objectives. Implementation strategies must account for heterogeneous sensitivity profiles. Countries with export-oriented agricultural systems require gradual transition timelines and enhanced market support to maintain food security and economic stability during dietary optimization processes (59–61).

### 4.4 Limitations and future directions

The methodology of this study is subject to several primary limitations. First, the reliance on PPP-adjusted producer prices, rather than retail consumer prices, prioritizes cross-country comparability within a production-side framework. An important limitation is that we use production costs as a proxy for consumer prices. Retail prices exhibit greater variability due to market dynamics, distribution systems, and processing costs. Future work should incorporate retail price data to better reflect consumer affordability (62). Second, the analysis focuses exclusively on optimizing animal product systems, without concurrently modeling plant-based production sectors. This targeted scope is justified by the unique paradox of ruminant livestock: while they are the primary drivers of agricultural greenhouse gas emissions, they remain nutritionally indispensable due to their biological efficiency. Mechanistic insights from microbiome research underscore this value, demonstrating how complex ruminal metabolic networks upcycle low-quality plant biomass into high-quality protein and essential micronutrients unattainable from crops alone (63, 64). The projected environmental benefits are not solely dependent on dietary shifts but also on the inherent efficiency of these livestock production systems. Feed efficiency gains refer to reducing the quantity of feed required per unit of animal product, thereby lowering resource inputs and environmental impacts associated with feed production. This distinction is crucial, as it implies that mitigation can be achieved through both consumer-side dietary changes and producer-side technical improvements. Consequently, while this study isolates the animal sector for optimization, future integrated models should synthesize these findings with plant-based system improvements to identify comprehensive food system transformation pathways. Furthermore, emerging protein sources, including cell-based meats and precision fermentation products, might provide alternative pathways to meet protein requirements with potentially lower environmental footprints, though their scalability and consumer acceptance remain uncertain. This scope reflects the pressing sustainability challenges posed by the concurrent expansion in both animal product production capacity and per capita consumption. Third, we acknowledge a limitation regarding the ‘input-efficiency' bias inherent in standard LCA methodologies. Our analysis relies on national emission factors that primarily reflect conventional production systems. Consequently, our model does not fully capture the system-specific environmental benefits of organic or regenerative agriculture, such as biodiversity enhancement and soil carbon sequestration (65). Recent evidence suggests that when soil carbon sequestration is accounted for, well-managed grass-fed systems can potentially act as net carbon sinks (66). Therefore, our results represent the environmental impacts of the current dominant production mix and may overestimate the emissions of systems employing regenerative grazing practices. The findings of this study highlight a critical tension in nutrition policy. Animal-sourced foods provide essential nutrients with superior bioavailability, including vitamin B12, complete amino acid profiles, and long-chain omega-3 fatty acids (67). However, current consumption levels in many countries exceed both nutritional requirements and environmental sustainability thresholds. The policy imperative is evident: a recalibration of animal product recommendations is necessary to ensure the maintenance of nutritional adequacy. This requires a systemic approach that transcends mere input-efficiency; it demands a fundamental restructuring of dietary patterns to constrain environmental impacts within the boundaries of the planet.

It is crucial to distinguish the scope of these findings. Our optimization pathways are specifically designed for high-income or rapidly transitioning economies that are currently grappling with overnutrition and high environmental footprints. We explicitly recognize that for many developing regions and food-insecure populations, increasing access to nutrient-dense animal-source foods remains critical for preventing stunting and cognitive malnutrition (40). Therefore, the reduction strategies proposed here should not be universally applied to the Global South, where baseline consumption is often below nutritional requirements.

In conclusion, moderate reductions in animal products, guided by nutrient-specific optimization, offer a viable strategy to reconcile nutritional adequacy, environmental sustainability, and economic feasibility. Context-specific policies are essential for enabling equitable and effective dietary transitions (68–72).

## 5 Conclusions

This study optimizes food-based dietary guidelines across four countries to reconcile nutritional adequacy, dietary costs, and environmental sustainability. Scenario analyses achieved greenhouse gas (GHG) emission reductions of 29–62% through strategic adjustments to animal product composition while maintaining isocaloric constraints. These findings emphasize the requirement for comprehensive nutritional frameworks that address multiple dietary components—including protein, micronutrients, and essential fatty acids—concurrently, rather than relying on single-nutrient optimization.

The feasibility of transitioning to sustainable diets varies significantly across nations. While these transitions may incur minimal additional costs in some high-income settings, they could increase dietary expenditure by 15% to 30% in other contexts, raising significant equity concerns and necessitating targeted policy intervention. Our results underscore that dietary guideline reforms depend heavily on the economic costs of production and the environmental footprint of locally produced animal products.

Countries with efficient livestock production systems and lower emission intensities face different optimization constraints than those with resource-intensive animal agriculture. Consequently, effective dietary transitions must be context-specific, integrating national production efficiencies with consumer affordability thresholds to support equitable and sustainable food system transformations.

## Data Availability

The original contributions presented in the study are included in the article/Supplementary material, further inquiries can be directed to the corresponding authors.
