# Supplementary material for: Multi-objective optimization of national dietary guidelines: balancing nutrition, environment, and economy
Source: Front Nutr. 2026 Mar 26;13:1758724. doi: 10.3389/fnut.2026.1758724 (PMC13061737; doi:10.3389/fnut.2026.1758724)
Supplement: Supplementary file 1 [file Table_1.docx]

Supplementary materials for:

**Multi-Objective Optimization of National Dietary Guidelines: Balancing Nutrition, Environment, and Economy**

Yehao Hu^†^ Fenghui Zhao^†^ Mengmeng Zhang Tingting Wang Han Li Tong Fu^*^ Tengyun Gao^*^ Chuanyou Su^*^

^1^ Henan International Joint Laboratory of Nutrition Regulation and Ecological Raising of Domestic Animal, College of Animal Science and Technology, Henan Agricultural University, Zhengzhou, China

*Corresponding author:

Email addresses: Chuanyou Su [(suchuanyou@henau.edu.cn)](mailto:(suchuanyou@henau.edu.cn))

Tong Fu (futong@henau.edu.cn)

Tengyun Gao (gaotengyun@henau.edu.cn)

In total 22 pages, 7 Note, 3 figure and 7 tables

Supplementary Note 1.

1. Sources and versions of current dietary guidelines in various countries

This study selected the current daily dietary guidelines of the United States (North America), Australia (Oceania), New Zealand (Southwest Pacific), and China (Eastern Asia) (Table S1), which contains the specific details of the national daily dietary guidelines currently implemented in the four countries, including the names and issuing units. These four countries were selected because they have clear daily recommended food intake guidelines and composition among all the countries that have issued dietary guidelines, and the dietary guidelines of the four countries, to our knowledge, have not been collated and similar nutrient optimization studies have been conducted.Furthermore, these countries represent different dietary patterns (Western vs. Asian), agricultural systems (intensive vs. mixed), and greenhouse gas emissions, allowing for a comparative analysis of sustainability trade-offs. Policymakers could implement differentiated subsidies (e.g., fish in China) or trade policies (e.g., importing low-GHG alternatives in the US) based on these findings.

1. Representation of foods in the dietary guidelines and conversion into product categories

The dietary guidelines of various countries only give the recommended food types. In our study, we set the recommended food types as specific food products and unified the representative products in the categories specified by each country. Although differences in culture and climate have caused differences in the output of food in various countries, the representative food products we set are all food products that are consumed daily in each country. In Table S2, we explain and set the food types involved in the dietary guidelines of various countries, converting the examples in the food groups in the dietary guidelines to the food in the daily food-based dietary guidelines.

1. Optimization plan settings for each country

In this study, there are generally five optimization schemes, namely, scaling animal product protein based on the FBDGs of each country according to the WHO recommended protein intake (MinProt scaled WHO), and then there are four schemes in two different contexts (MaxVitamins scaled FBDGs, MaxVitamins scaled WHO, MaxFat scaled FBDGs, MaxFat scaled WHO). In Table S3, we list the details of animal and plant products in the MinProt scaled WHO scheme of the four countries.

1. Calculation method of greenhouse gas emissions for each optimization scheme

The greenhouse gas emissions of the optimization schemes of each country are based on the sum of the greenhouse gas emissions of animal products produced in each country. The individual situations of each animal product in the context of imbalance in each country are balanced by each country's own research. The selection of articles based on the same LCA model and the use of national data of the entire industry chain is prioritized. In terms of time, we select the last article as a representative. If there are multiple articles with data, we take the average value of the greenhouse gas emissions of the product into our study (see Table S4 for selected articles and values).

1. Calculate the nutrient contribution of animal products in each allocation scenario

To quantify the inherent nutritional contributions of animal-source foods (ASFs) under each country’s dietary guidelines, we calculated the baseline nutrient contribution of each food item using standardized nutrient composition data combined with country-specific food allocations. Nutrient composition values for all foods were obtained from the USDA FoodData Central Standard Reference Legacy database (2018 [FNDDS Flavonoid database : USDA ARS](https://www.ars.usda.gov/northeast-area/beltsville-md-bhnrc/beltsville-human-nutrition-research-center/food-surveys-research-group/docs/fndds-flavonoid-database/)) Values originally reported per 100 g edible portion were converted to per-kilogram edible weight to ensure consistency across food groups.

Let *i* index animal-source foods (ASF) categories (milk, eggs, poultry, pork, beef, fish) and *n* index nutrients. For each country, the baseline daily allocation of food item *i* is denoted as *x_i_* (kg/person/day). The edible portion coefficient is denoted as *e_i_* (unitless; where data were already reported on an edible-weight basis, *e_i_*=1). Nutrient density for nutrient nnn in food *i* is denoted as *N_i,n_* (expressed in g or mg per kg edible weight, matched to the unit of the corresponding reference requirement). When applicable, a nutrient-specific bioavailability factor *B_i,n_* was applied; in cases where reliable estimates were unavailable or unnecessary, *B_i,n_* was set to 1.

The baseline nutrient contribution of food item *i* to nutrient *n* was calculated as:

__ (Formula S5.1)

The total baseline contribution of all ASFs to nutrient *n* for a given country is:

__ (Formula S5.2)

where *A* denotes the set of all ASFs included in this study.

Reference nutrient intake values *R_n_* were compiled from authoritative international recommendations and peer-reviewed literature. For nutrients lacking explicit consensus values, the most recent recommendations from recognized bodies were used (see Appendix S6 for detailed sources). All units for nutrient densities, intakes, and reference requirements were harmonized to ensure internal consistency.

This framework provides a standardized and transparent baseline for quantifying nutrient provision from animal-source foods prior to implementing the optimization scenarios described in the main text.

1. Determination of nutrient requirements

The nutritional supply of each country's FBDGs is calculated based on the composition of each country's food products and our nutritional value reference. The nutrient requirements for the five scenarios we formulated are based on surveys by various organizations (Table S6). Basically, the content of the survey is based on global nutritional levels. Selecting and using such data may provide fairer nutrient requirement values for the proposals.The age range we considered was 14 years and above, excluding special periods (such as pregnancy and breastfeeding), because during this period, nutrient requirements need to be considered again.

Regarding the selected values of vitamins, we averaged the minimum recommended dietary intake and the maximum recommended dietary intake according to age standards to achieve universality. Regarding the selected values of fatty acids, we did not use a general average, but a higher value, because our research is to reduce the amount of animal products, which are the main suppliers of fatty acids in the dietary guidelines. Because our research goal is to optimize the intake of animal products, which are the main source of fatty acids in the dietary guidelines, we hope to use a higher value to explore which animal products will contribute to the nutritional contribution of fatty acid nutrients in the diet when animal product intake is reduced, thereby providing direction and insights for subsequent research.

**Table S1.** Overview of food-based dietary guidelines employed.

| **Country** | **Food-based dietary guideline** | **Responsible institution** |
| --- | --- | --- |
| **Australia** | Australian Dietary Guidelines Summary | National Health and Medical Research Council |
| **China** | Dietary Guidelines for Chinese Residents | Chinese Nutrition Society |
| **United States** | Dietary Guidelines for Americans 2020—2025 | U.S.Department of Agriculture |
| **New Zealand** | Eating and Activity Guidelines | Health New Zealand |


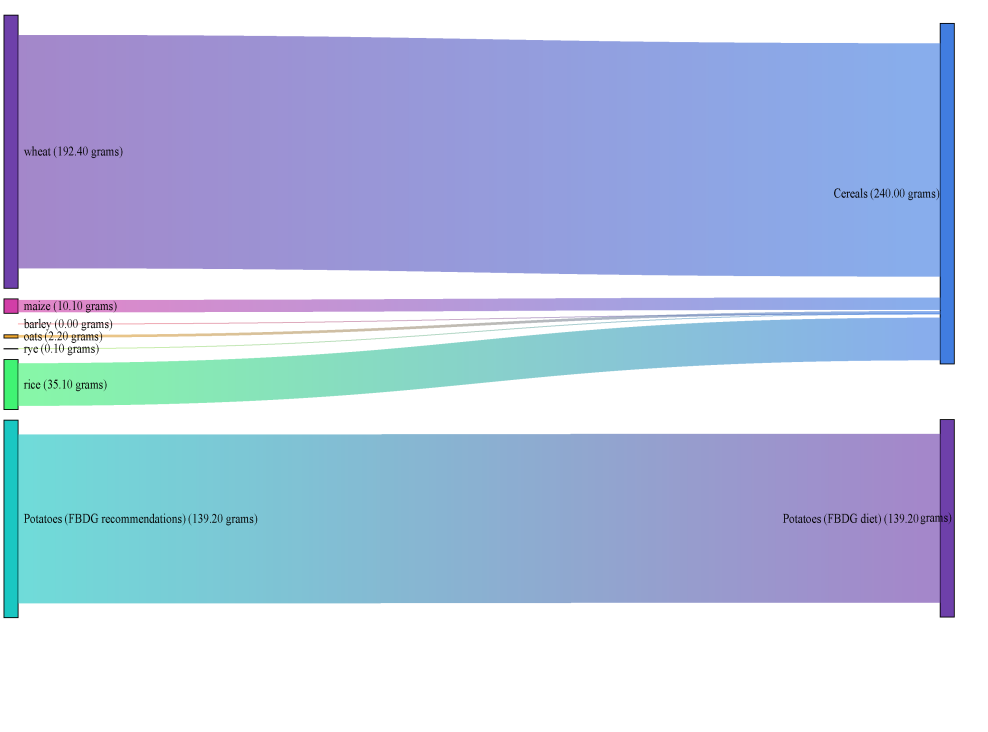


**Figure S1. Translating dietary guidelines into daily diet**

Process of translating FBDG recommendations (left) into daily FBDG diets (right) with fractions of the Food Balance Sheet data (middle), taking the example of the food group “grains” of the Dutch dietary guidelines. FBDG: food-based dietary guideline.

**Table S2.**  Division of food groups into food items. FBDG: food-based dietary guideline.

| **Food group** | **Food item examples in FBDGs** | **Food items in the daily FBDG diets** |
| --- | --- | --- |
| **Grains** | Pasta, rice, couscous, breakfast cereals, flour from grains (all limitedly processed and wholegrain) | Wheat, maize, barley, oats, rye, rice |
| **Bread** | Whole wheat bread | Whole wheat bread |
| **Potatoes** | Potatoes | Potato, sweet potato |
| **Vegetables** | All vegetables (fresh or frozen) | Tomatoes, onions, other vegetables |
| **Fruits** | All fruits (fresh, dried or frozen) | Apples, bananas, oranges and mandarins, lemons, limes and products, grapefruit and products, citrus other, plantains, pineapple and products, dates (dried), grapes and products, fruits other |
| **Legumes** | All pulses (Fresh or canned without salt) | Beans, soybeans, pulses other and products |
| **Dairy^1^** | Milk, yoghurt, cheese  (low-fat, unflavoured and/or low salt) | Milk, yoghurt, cheese |
| **Red meat** | Beef, pork (unprocessed) | Bovine meat, pork |
| **White meat** | Chicken, turkey (unprocessed) | Chicken |
| **Fish** | All types of fish (emphasis on fatty fish) | Freshwater fish, marine fish, aquaculture (salmon, tilapia) |
| **Eggs** | Eggs | Eggs |
| **Nuts** | All nuts (unroasted and unsalted) | Mixed nuts and products, peanuts |
| **Seeds** | All seeds (unsalted) | Sunflower seeds, sesame seeds |
| **Oils and fats** | All vegetable oils (emphasis on olive oil and flaxseed oil) | Peanut oil, sunflower oil, soybean oil, vegetable oil, olive oil,rapeseed oil, oilcrops other |
| **Beverages**^2^ | Water, coffee, tea, fresh fruit juice, alcoholic beverages (without added sugar) | Coffee, tea, fruit juice |

^1^Recommendations for cheese and/or yoghurt were partly provided separately, and therefore not determined via the Food Balance Sheet shares.

^2^Not all FBDGs included coffee, tea, and fruit juice.

**Table S3 Daily dietary guidelines and protein restriction plans for each country (according to the WHO recommendation of 60 g of protein per day)**

| **Country** | **Food group** | **FBDG** | **MinProt**  **(scaled WHO)** |
| --- | --- | --- | --- |
| China | Milk | 300 | 207.8 |
| China | Beef | 16 | 13.4 |
| China | Pork | 21.2 | 13.4 |
| China | Chicken | 14.8 | 20.1 |
| China | Eggs | 46 | 31.9 |
| China | Fish | 58 | 40.2 |
| China | Cereals | 350 | 242.5 |
| China | Potatoes | 75 | 52.0 |
| China | Vegetables | 630 | 436.5 |
| China | Legumes and nuts | 29 | 20 |
| China | Oils and fats | 27 | 10.7 |
| China | Fruits | 270 | 187.2 |
| New Zealand | Milk | 625 | 427.9 |
| New Zealand | Beef | 12.8 | 8.8 |
| New Zealand | Pork | 12.8 | 8.8 |
| New Zealand | Chicken | 31.5 | 21.6 |
| New Zealand | Eggs | 47.2 | 32.3 |
| New Zealand | Fish | 39.3 | 26.9 |
| New Zealand | Cereals | 240 | 164.4 |
| New Zealand | Potatoes | 118.8 | 201.5 |
| New Zealand | Vegetables | 294.2 | 272.2 |
| New Zealand | Legumes and nuts | 8.02 | 8.1 |
| New Zealand | Oils and fats | 27 | 8.4 |
| New Zealand | Fruits | 300 | 205.3 |
| Australia | Milk | 625 | 445.9 |
| Australia | Beef | 42.8 | 9.1 |
| Australia | Pork | 36 | 9.1 |
| Australia | Chicken | 68.8 | 22.5 |
| Australia | Eggs | 11.3 | 33.7 |
| Australia | Fish | 8.9 | 28.0 |
| Australia | Cereals | 240 | 171.3 |
| Australia | Potatoes | 139.2 | 99.3 |
| Australia | Vegetables | 273.8 | 286.3 |
| Australia | Legumes and nuts | 11.3 | 8.4 |
| Australia | Oils and fats | 27 | 1.7 |
| Australia | Fruits | 300 | 214 |
| U.S.A | Milk | 710 | 502.1 |
| U.S.A | Beef | 17.9 | 12.7 |
| U.S.A | Pork | 17.9 | 12.7 |
| U.S.A | Chicken | 35.7 | 25.2 |
| U.S.A | Eggs | 35.7 | 25.2 |
| U.S.A | Fish | 35 | 24.7 |
| U.S.A | Cereals | 426 | 128.8 |
| U.S.A | Potatoes | 188.5 | 133.3 |
| U.S.A | Vegetables | 616 | 435.5 |
| U.S.A | Legumes and nuts | 19 | 13.5 |
| U.S.A | Oils and fats | 26.6 | 15.2 |
| U.S.A | Fruits | 426 | 301.1 |

**Table S4 Greenhouse gas emissions from animal products in daily diets in different countries**

| **Country** | **Item** | **GHG**^1^ | **References** |
| --- | --- | --- | --- |
| Australia | Milk production | 1.11 CO_2_^-e^/kg 1 FPCM^2^ | (Gollnow et al., 2014) |
| United States | Milk production | 0.92 CO_2_^-e^/kg 1 FPCM | (Mazzetto et al., 2020) |
| China | Milk production | 2.00 CO_2_^-e^/kg 1 FPCM | (Wang et al., 2016)**、**(Wei et al., 2024) |
| New Zealand | Milk production | 1.10 CO_2_^-e^/kg 1 FPCM | (Flysjö et al., 2011)、(Ledgard et al., 2007) |
| Australia | Beef production | 31.28 CO_2_^-e^/kg 1 kg LW^3^ | (Wiedemann et al., 2015)、(Peters et al., 2010) |
| United States | Beef production | 27.05 CO_2_^-e^/kg 1 kg LW | (Lupo et al., 2013)、(Tichenor et al., 2017) |
| China | Beef production | 45.58 CO_2_^-e^/kg 1 kg LW | (Wei et al., 2023) |
| New Zealand | Beef production | 53.84 CO_2_^-e^/kg 1 kg LW | (van Selm et al., 2021)、(Payen et al., 2020) |
| Australia | Pork production | 3.70 CO_2_^-e^/kg 1 kg LW | (Wiedemann et al., 2018)、(Copley et al., 2024) |
| United States | Pork production | 4.46 CO_2_^-e^/kg 1 kg LW | (Pelletier et al., 2010)、(Stone et al., 2012) |
| China | Pork production | 1.90 CO_2_^-e^/kg 1 kg LW | (Long et al., 2021) |
| New Zealand | Pork production | 4.87 CO_2_^-e^/kg 1 kg LW | (Philippe and Nicks, 2015) |
| Australia | Fish production | 1.00 CO_2_^-e^/kg 1 kg fish | (A. K. Farmery et al., 2015)、(Almeida et al., 2014)、(A. Farmery et al., 2015)、(Jiang et al., 2022) |
| United States | Fish production | 8.50 CO_2_^-e^/kg 1 kg fish | (Hollmann, 2017)、(Jiang et al., 2022) |
| China | Fish production | 5.73 CO_2_^-e^/kg 1 kg fish | (Xing-Guo et al., 2023)、(Jiang et al., 2022) |
| New Zealand | Fish production | 3.04 CO_2_^-e^/kg 1 kg fish | (Jiang et al., 2022)、(Sonesson et al., 2010) |
| Australia | Chicken production | 2.84 CO_2_^-e^/kg 1 kg LW | (Wiedemann et al., 2017)、(Bengtsson and Seddon, 2013) |
| United States | Chicken production | 2.81 CO_2_^-e^/kg 1 kg LW | (Pelletier, 2008) |
| China | Chicken production | 7.44 CO_2_^-e^/kg 1 kg LW | (Cheng et al., 2023) |
| New Zealand | Chicken production | 3.97 CO_2_^-e^/kg 1 kg LW | (Hoolohan et al., 2013) |
| Australia | Egg production | 2.87 CO_2_^-e^/kg 1 kg eggs | (Copley et al., 2023) |
| United States | Egg production | 2.10 CO_2_^-e^/kg 1 kg eggs | (Pelletier et al., 2014) |
| China | Egg production | 3.58 CO_2_^-e^/kg 1 kg eggs | (Luo et al., 2015) |
| New Zealand | Egg production | 4.93 CO_2_^-e^/kg 1 kg eggs | (Williams et al., n.d.) |

^1^ Greenhouse gas emissions per unit of animal product production.

^2^ FPCM : Fat/protein-corrected milk production

^3^ LW : Live weight

**Table S5** Minimum and maximum intake of foods or food groups based on the EAT–Lancet dietary guidelines. All mass units are in fresh weights

| **Item** | **Minimum** | **Maximum** | **Unit** |
| --- | --- | --- | --- |
| **Grains** | 0 | 60 | % of energy uptake |
| **Tubers** | 0 | 100 | g per day per capita |
| **Vegetables** | 200 | 600 | g per day per capita |
| **Fruit** | 100 | 300 | g per day per capita |
| **Dairy** | 0 | 500 | g per day per capita |
| **Red meat** | 0 | 28 | g per day per capita |
| **Chicken** | 0 | 58 | g per day per capita |
| **Eggs** | 0 | 25 | g per day per capita |
| **Fish** | 0 | 100 | g per day per capita |
| **Legumes** | 0 | 225 | g per day per capita |
| **Nuts/seeds** | 0 | 75 | g per day per capita |
| **Sugar** | 0 | 31 | g per day per capita |

**Table S6. Sources of recommended nutrient values for optimization plans**

| **Item** | **Minimum** | **Maximum** | **Selected Value** | **References** |
| --- | --- | --- | --- | --- |
| **Vitamin A** | 700 mcg RAE | 900 mcg RAE | 800 mcg RAE | USPSTF(Jin, 2022a),(Institute of Medicine (US) Panel on Micronutrients, 2001) |
| **Vitamin B6** | 1.2 mg | 1.7 mg | 1.45 mg | (Jin, 2022b) |
| **Vitamin B12** | 2.4 mcg | 2.4 mcg | 2.4 mcg | (Institute of Medicine (US), 1998) |
| **EPA+DHA** | 100 mg | 500 mg | 400 mg | (Koletzko et al., 2014),(Kris-Etherton et al., 2002),(EFSA Panel on Dietetic Products, Nutrition and Allergies (NDA), 2009) |
| **ALA** | 1.1 g | 1.6 g | 1.6 g | (Trumbo et al., 2002) |


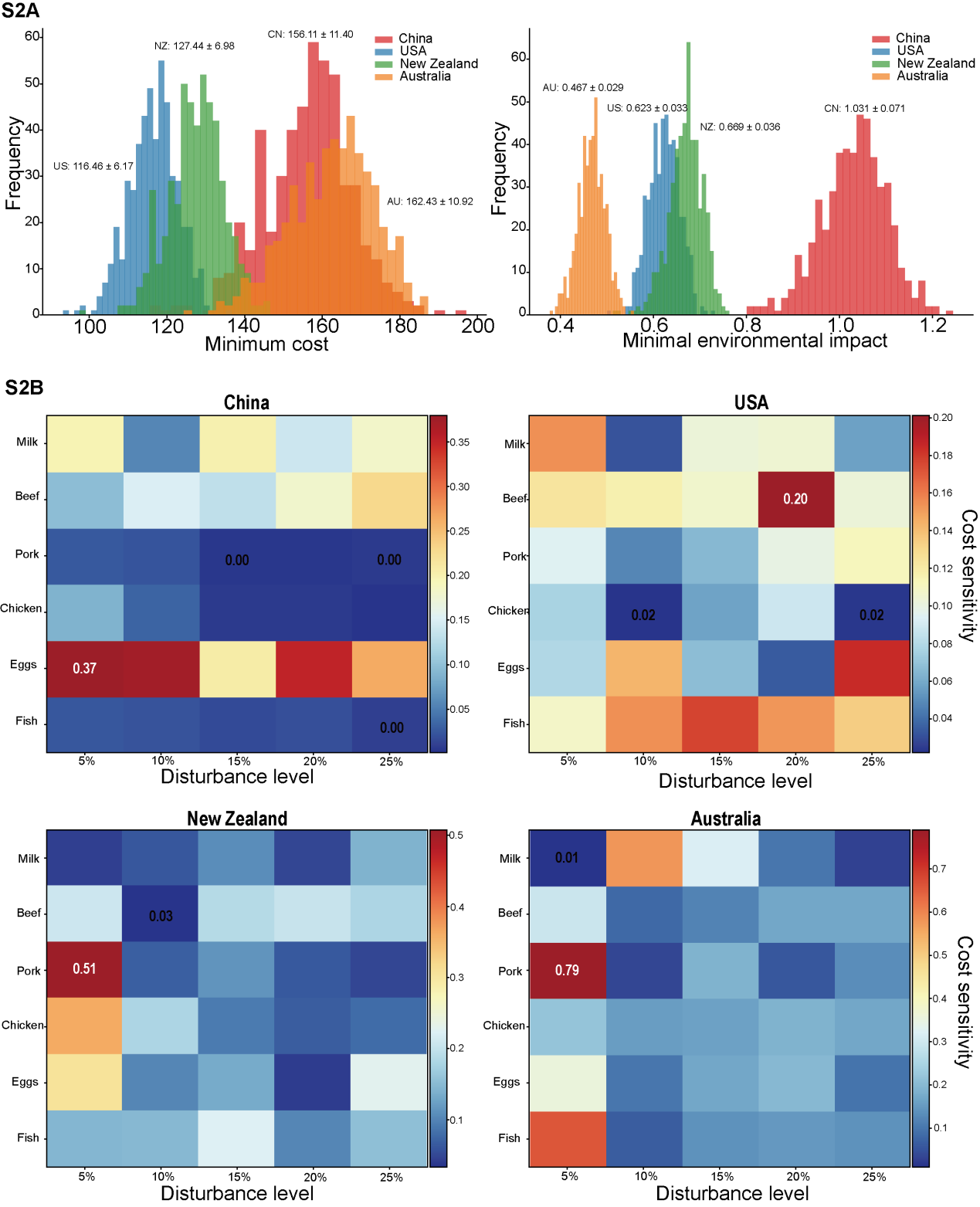


**Figure S2 Uncertainty analysis and sensitivity analysis**

S2A shows the uncertainty analysis results of the minimum environmental impact and minimum economic impact, and S2B is a heat map of economic sensitivity, representing the economic sensitivity of each food in each country under different disturbance levels.


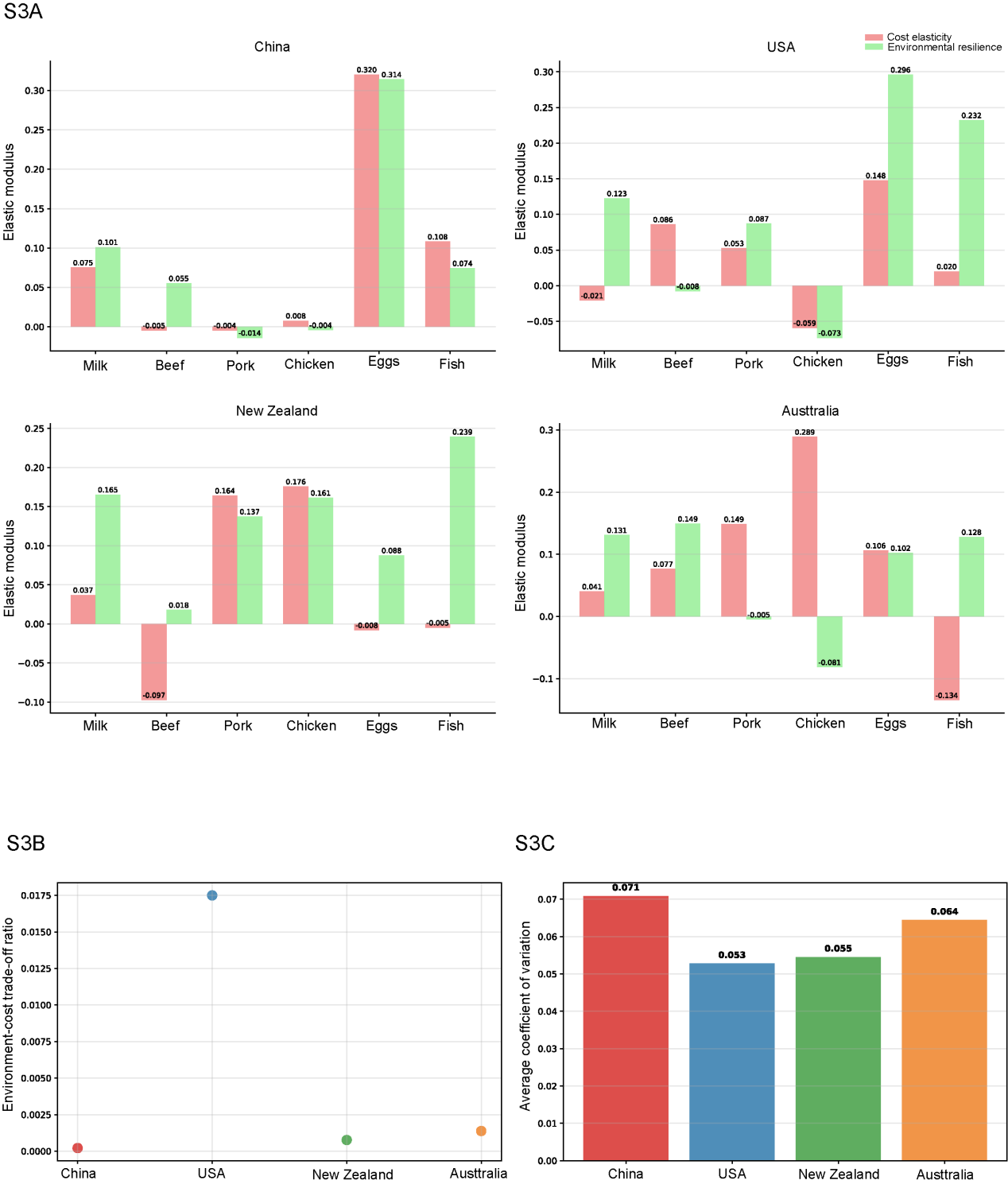


**Figure S3 Response analysis and robustness analysis**

S3A shows the sensitivity results for environmental and economic impacts, illustrating the economic and environmental elasticities of each country when the optimal solution is perturbed by ±20%. S3B shows the stability of the cost-environmental trade-off, and S3C represents the average coefficient of variation of the economic-environmental response under the average level of uncertainty.

**REFERENCES**

Almeida, C., Vaz, S., Cabral, H., Ziegler, F., 2014. Environmental assessment of sardine (Sardina pilchardus) purse seine fishery in Portugal with LCA methodology including biological impact categories. Int J Life Cycle Assess 19, 297–306. https://doi.org/10.1007/s11367-013-0646-5

Bengtsson, J. & Seddon, J. Cradle to retailer or quick service restaurant gate life cycle assessment of chicken products in Australia. *Journal of Cleaner Production* **41**, 291–300 (2013). https://doi.org/10.1016/j.jclepro.2012.09.034

Cheng, Z., Jia, Y., Bai, Y., Zhang, T., Ren, K., Zhou, X., Zhai, Y., Shen, X., Hong, J., 2023. Intensifying the environmental performance of chicken meat production in China: From perspective of life cycle assessment. Journal of Cleaner Production 384, 135603. https://doi.org/10.1016/j.jclepro.2022.135603

Copley, M.A., McGahan, E.J., McCormack, K., Wiedemann, S.G., 2024. Environmental impacts of Australian pork in 2020 and 2022 determined using lifecycle assessments. Anim. Prod. Sci. 64. https://doi.org/10.1071/AN23352

Copley, M.A., Wiedemann, S.G., McGahan, E.J., 2023. Environmental impacts of the Australian poultry industry. 2. Egg production. Anim. Prod. Sci. 63, 505–521. https://doi.org/10.1071/AN22297

EFSA Panel on Dietetic Products, Nutrition and Allergies (NDA), 2009. Scientific Opinion on the substantiation of health claims related to EPA, DHA, DPA and maintenance of normal blood pressure (ID 502), maintenance of normal HDL-cholesterol concentrations (ID 515), maintenance of normal (fasting) blood concentrations of triglycerides (ID 517), maintenance of normal LDL-cholesterol concentrations (ID 528, 698) and maintenance of joints (ID 503, 505, 507, 511, 518, 524, 526, 535, 537) pursuant to Article 13(1) of Regulation (EC) No 1924/2006. EFSA Journal 7, 1263. https://doi.org/10.2903/j.efsa.2009.1263

Farmery, A., Gardner, C., Green, B. S., Jennings, S. & Watson, R. Life cycle assessment of wild capture prawns: expanding sustainability considerations in the Australian Northern Prawn Fishery. *Journal of Cleaner Production* **87**, 96–104 (2015). https://doi.org/10.1016/j.jclepro.2014.10.063

Farmery, A. K., Gardner, C., Green, B. S., Jennings, S. & Watson, R. A. Domestic or imported? An assessment of carbon footprints and sustainability of seafood consumed in Australia. *Environmental Science & Policy* **54**, 35–43 (2015). https://doi.org/10.1016/j.envsci.2015.06.007

Flysjö, A., Henriksson, M., Cederberg, C., Ledgard, S. & Englund, J.-E. The impact of various parameters on the carbon footprint of milk production in New Zealand and Sweden. *Agricultural Systems* **104**, 459–469 (2011).

Gollnow, S. *et al.* Carbon footprint of milk production from dairy cows in Australia. *International Dairy Journal* **37**, 31–38 (2014). https://doi.org/10.1016/j.idairyj.2014.02.005

Hollmann, R., 2017. An Aquaponics Life Cycle Assessment: Evaluating an Inovative Method for Growing Local Fish and Lettuce.

Hoolohan, C., Berners-Lee, M., McKinstry-West, J. & Hewitt, C. N. Mitigating the greenhouse gas emissions embodied in food through realistic consumer choices. *Energy Policy* **63**, 1065–1074 (2013). https://doi.org/10.1016/j.enpol.2013.09.046

Institute of Medicine (US) Panel on Micronutrients, 2001. Dietary Reference Intakes for Vitamin A, Vitamin K, Arsenic, Boron, Chromium, Copper, Iodine, Iron, Manganese, Molybdenum, Nickel, Silicon, Vanadium, and Zinc. National Academies Press (US), Washington (DC). https://doi.org/10.1016/s0002-8223(02)90346-9

Institute of Medicine (US) Standing Committee on the Scientific Evaluation of Dietary Reference Intakes and its Panel on Folate, Other B Vitamins, and Choline, 1998. Dietary Reference Intakes for Thiamin, Riboflavin, Niacin, Vitamin B6, Folate, Vitamin B12, Pantothenic Acid, Biotin, and Choline, The National Academies Collection: Reports funded by National Institutes of Health. National Academies Press (US), Washington (DC). https://doi.org/10.17226/6015

Jiang, Q., Bhattarai, N., Pahlow, M. & Xu, Z. Environmental sustainability and footprints of global aquaculture. *Resources, Conservation and Recycling* **180**, 106183 (2022). https://doi.org/10.1016/j.resconrec.2022.106183

Jin, J., 2022a. Vitamins and Minerals to Prevent Cardiovascular Disease and Cancer. JAMA 327, 2364. https://doi.org/10.1001/jama.2022.10009

Jin, J., 2022b. Vitamins and Minerals to Prevent Cardiovascular Disease and Cancer. JAMA 327, 2364. https://doi.org/10.1001/jama.2022.10009

Koletzko, B., Boey, C.C.M., Campoy, C., Carlson, S.E., Chang, N., Guillermo-Tuazon, M.A., Joshi, S., Prell, C., Quak, S.H., Sjarif, D.R., Su, Y., Supapannachart, S., Yamashiro, Y., Osendarp, S.J.M., 2014. Current information and Asian perspectives on long-chain polyunsaturated fatty acids in pregnancy, lactation, and infancy: systematic review and practice recommendations from an early nutrition academy workshop. Ann Nutr Metab 65, 49–80. https://doi.org/10.1159/000365767

Kris-Etherton, P.M., Harris, W.S., Appel, L.J., American Heart Association. Nutrition Committee, 2002. Fish consumption, fish oil, omega-3 fatty acids, and cardiovascular disease. Circulation 106, 2747–2757. https://doi.org/10.1161/01.cir.0000038493.65177.94

Ledgard, S.F., Basset-Mens, C., Mclaren, S., Boyes, M., 2007. Energy use, “food miles” and greenhouse gas emissions from New Zealand dairying - how efficient are we? Proceedings of the New Zealand Grassland Association 223–228. https://doi.org/10.33584/jnzg.2007.69.2665

Long, W., Wang, H., Hou, Y., Chadwick, D., Ma, Y., Cui, Z., Zhang, F., 2021. Mitigation of Multiple Environmental Footprints for China’s Pig Production Using Different Land Use Strategies. Environ. Sci. Technol. 55, 4440–4451. https://doi.org/10.1021/acs.est.0c08359

Luo, T., Yue, Q., Yan, M., Cheng, K., Pan, G., 2015. Carbon footprint of China’s livestock system – a case study of farm survey in Sichuan province, China. Journal of Cleaner Production 102, 136–143. https://doi.org/10.1016/j.jclepro.2015.04.077

Lupo, C.D., Clay, D.E., Benning, J.L., Stone, J.J., 2013. Life-cycle assessment of the beef cattle production system for the northern great plains, USA. J Environ Qual 42, 1386–1394. https://doi.org/10.2134/jeq2013.03.0101

Mazzetto, A. M. *et al.* Comparing the environmental efficiency of milk and beef production through life cycle assessment of interconnected cattle systems. *Journal of Cleaner Production* **277**, 124108 (2020). https://doi.org/10.1016/j.jclepro.2020.124108

Payen, S., Falconer, S., Carlson, B., Yang, W. & Ledgard, S. Eutrophication and climate change impacts of a case study of New Zealand beef to the European market. *Science of The Total Environment* **710**, 136120 (2020). https://doi.org/10.1016/j.scitotenv.2019.136120

Pelletier, N. Environmental performance in the US broiler poultry sector: Life cycle energy use and greenhouse gas, ozone depleting, acidifying and eutrophying emissions. *Agricultural Systems* **98**, 67–73 (2008). https://doi.org/10.1016/j.agsy.2008.03.007

Pelletier, N., Ibarburu, M. & Xin, H. Comparison of the environmental footprint of the egg industry in the United States in 1960 and 2010 1. *Poultry Science* **93**, 241–255 (2014). https://doi.org/10.3382/ps.2013-03390

Pelletier, N., Lammers, P., Stender, D. & Pirog, R. Life cycle assessment of high- and low-profitability commodity and deep-bedded niche swine production systems in the Upper Midwestern United States. *Agricultural Systems* **103**, 599–608 (2010). https://doi.org/10.1016/j.agsy.2010.07.001

Peters, G.M., Rowley, H.V., Wiedemann, S., Tucker, R., Short, M.D., Schulz, M., 2010. Red Meat Production in Australia: Life Cycle Assessment and Comparison with Overseas Studies. Environ. Sci. Technol. 44, 1327–1332. https://doi.org/10.1021/es901131e

Philippe, F.-X. & Nicks, B. Review on greenhouse gas emissions from pig houses: Production of carbon dioxide, methane and nitrous oxide by animals and manure. *Agriculture, Ecosystems & Environment* **199**, 10–25 (2015). https://doi.org/10.1016/j.agee.2014.08.015

Sonesson, U., Davis, J., Ziegler, F., 2010. Food production and emissions of greenhouse gases: an overview of the climate impact of different product groups.

Stone, J. J., Dollarhide, C. R., Benning, J. L., Gregg Carlson, C. & Clay, D. E. The life cycle impacts of feed for modern grow-finish Northern Great Plains US swine production. *Agricultural Systems* **106**, 1–10 (2012). https://doi.org/10.1016/j.agsy.2011.11.002

Tichenor, N. E., Peters, C. J., Norris, G. A., Thoma, G. & Griffin, T. S. Life cycle environmental consequences of grass-fed and dairy beef production systems in the Northeastern United States. *Journal of Cleaner Production* **142**, 1619–1628 (2017). https://doi.org/10.1016/j.jclepro.2016.11.138

Trumbo, P., Schlicker, S., Yates, A.A., Poos, M., Food and Nutrition Board of the Institute of Medicine, The National Academies, 2002. Dietary reference intakes for energy, carbohydrate, fiber, fat, fatty acids, cholesterol, protein and amino acids. J Am Diet Assoc 102, 1621–1630. https://doi.org/10.1016/s0002-8223(02)90346-9

Sebastian RS, Goldman JD, Martin CL, Steinfeldt LC, Wilkinson Enns C, Moshfegh AJ. 2014 (slightly revised Dec. 2015). Flavonoid Values for USDA Survey Foods and Beverages 2007-2008. Beltsville, MD: U.S. Department of Agriculture, Agricultural Research Service, Food Surveys Research Group. Available from:www.ars.usda.gov/nea/bhnrc/fsrg

van Selm, B., de Boer, I. J. M., Ledgard, S. F. & van Middelaar, C. E. Reducing greenhouse gas emissions of New Zealand beef through better integration of dairy and beef production. *Agricultural Systems* **186**, 102936 (2021). https://doi.org/10.1016/j.agsy.2020.102936

Wang, X., Kristensen, T., Mogensen, L., Knudsen, M. T. & Wang, X. Greenhouse gas emissions and land use from confinement dairy farms in the Guanzhong plain of China – using a life cycle assessment approach. *Journal of Cleaner Production* **113**, 577–586 (2016). https://doi.org/10.1016/j.jclepro.2015.11.099

Wei, S., Ledgard, S., Fan, J., Tian, Y. & Dong, H. Carbon footprints, mitigation effects and economic performance of dairy farm systems in Inner Mongolia. *Agricultural Systems* **214**, 103835 (2024). https://doi.org/10.1016/j.agsy.2023.103835

Wei, Y., Zhang, X., Xu, M. & Chang, Y. Greenhouse gas emissions of meat products in China: A provincial-level quantification. *Resources, Conservation and Recycling* **190**, 106843 (2023). https://doi.org/10.1016/j.resconrec.2022.106843

Wiedemann, S., McGahan, E., Murphy, C., Yan, M., 2015. Resource use and environmental impacts from beef production in eastern Australia investigated using life cycle assessment. Anim. Prod. Sci. 56, 882–894. https://doi.org/10.1071/AN14687

Wiedemann, S.G., McGahan, E.J., Murphy, C.M., 2018. Environmental impacts and resource use from Australian pork production determined using life cycle assessment. 2. Energy, water and land occupation. Anim. Prod. Sci. 58, 1153. https://doi.org/10.1071/AN16196

Wiedemann, S. G., McGahan, E. J. & Murphy, C. M. Resource use and environmental impacts from Australian chicken meat production. *Journal of Cleaner Production* **140**, 675–684 (2017). https://doi.org/10.1016/j.jclepro.2016.06.086

Williams, A., Audsley, E., Jones, R., Weller, R., Bryson, R., Philipps, L., Whitmore, A., Glendining, M., Dailey, G., n.d. Determining the environmental burdens and resource use in the production of agricultural and horticultural commodities. Defra project report IS0205. https://doi.org/10.1533/9780857090225.2.98

Xing-Guo, L., Hong-ye, S., Zhao-jun, G., Guofeng, C., Jie, W., Hao, Z., 2023. The environmental impact and development direction of grass carp, Ctenopharyngodon idella, aquaculture. Journal of the World Aquaculture Society 54, 1354–1366. https://doi.org/10.1111/jwas.12990
